# Comparative gene expression in toxic versus non-toxic strains of the marine dinoflagellate *Alexandrium minutum*

**DOI:** 10.1186/1471-2164-11-248

**Published:** 2010-04-19

**Authors:** Ines Yang, Uwe John, Sára Beszteri, Gernot Glöckner, Bernd Krock, Alexander Goesmann, Allan D Cembella

**Affiliations:** 1 Alfred-Wegener-Institut für Polar-und Meeresforschung, Am Handelshafen 12, 27570 Bremerhaven, Germany; 2 School of Biosciences, University of Exeter, Stocker Road, Exeter, EX4 4QD, UK; 3 Fritz Lipmann Institute for Age Research, Beutenbergstraß 11, 07745 Jena, Germany; 4 Institute for Biochemistry I, University of Cologne, Joseph-Stelzmann-Str. 52, 50931 Cologne, Germany; 5 Berlin Center for Genomics in Biodiversity Research, Königin-Luise-Str. 1-3, 14195 Berlin, Germany; 6 Universität Bielefeld, CeBiTec, 33594 Bielefeld, Germany

## Abstract

**Background:**

The dinoflagellate *Alexandrium minutum *typically produces paralytic shellfish poisoning (PSP) toxins, which are known only from cyanobacteria and dinoflagellates. While a PSP toxin gene cluster has recently been characterized in cyanobacteria, the genetic background of PSP toxin production in dinoflagellates remains elusive.

**Results:**

We constructed and analysed an expressed sequence tag (EST) library of *A. minutum*, which contained 15,703 read sequences yielding a total of 4,320 unique expressed clusters. Of these clusters, 72% combined the forward-and reverse reads of at least one bacterial clone. This sequence resource was then used to construct an oligonucleotide microarray. We analysed the expression of all clusters in three different strains. While the cyanobacterial PSP toxin genes were not found among the *A. minutum *sequences, 192 genes were differentially expressed between toxic and non-toxic strains.

**Conclusions:**

Based on this study and on the lack of identified PSP synthesis genes in the two existent *Alexandrium tamarense *EST libraries, we propose that the PSP toxin genes in dinoflagellates might be more different from their cyanobacterial counterparts than would be expected in the case of a recent gene transfer. As a starting point to identify possible PSP toxin-associated genes in dinoflagellates without relying on *a priori *sequence information, the sequences only present in mRNA pools of the toxic strain can be seen as putative candidates involved in toxin synthesis and regulation, or acclimation to intracellular PSP toxins.

## Background

*Alexandrium minutum *is a bloom-forming toxic dinoflagellate typically capable of producing paralytic shellfish poisoning (PSP) toxins. Occurring from northern Europe and the Mediterranean to Asia, Australia and New Zealand, *A. minutum *poses a widespread threat to seafood production and consumer health (for a review of biogeography and species boundaries see [1]). PSP is potentially fatal to both humans [2] and marine fauna, particularly vertebrates [3,4]; human intoxications typically occur after ingestion of suspension-feeding bivalve molluscs.

PSP toxins are tetrahydropurine neurotoxins, which specifically bind to voltage-gated sodium channels of nerves and muscle cells. The most well known representative, saxitoxin, is probably also the parent compound [5], of which more than 20 naturally occurring derivatives are known  [6], including the gonyautoxins produced by *A. minutum*. Several physiological or ecological functions of PSP toxins have been considered (reviewed in [7]), among others chemical defence [7], nitrogen storage [7], activity as pheromones [8], and influences on the dinoflagellate-associated bacteria in the phycosphere [9]. Several experimental studies link PSP toxicity in dinoflagellates to deterring effects on some species of copepod grazers [10,11], which can lead to a redirection of grazing pressure onto non-toxic phytoplankton species competing for the same nutrient resources [12], but the interactions and deterrence responses are highly species-specific and not universal.

In addition to these effects, but independent of PSP toxin content, several *Alexandrium *species including *A. minutum *were shown to produce lytic substances which affect both competing algae and unicellular predators [13,14]. Both these factors potentially contribute to the formation and maintenance of *A. minutum *blooms.

Analytical and physical-chemical methods can resolve structures and provide basic biosynthetic pathways to phycotoxins, but provide little direct evidence on specific biosynthetic enzymes and regulatory functions [15]. Thus, a combined approach linked to genomics and proteomics is required to fully describe toxin biosynthesis and regulation. In the case of PSP toxins in dinoflagellates, however, this approach is rather challenging, as both the physiological regulation and evolutionary origin of PSP toxins remain enigmatic.

Apart from marine dinoflagellates, comprising several species of *Alexandrium *and a single species *Pyrodinium bahamense *of a closely related genus, plus *Gymnodinium catenatum*, which belongs to a very distantly related phylogenetic group (see [16,17]), the only confirmed PSP toxin producers are predominantly freshwater cyanobacteria, including members of the genera *Cylindrospermopsis*, *Anabaena*, *Lyngbya *and *Aphanizomenon*. This paraphyletic distribution was confirmed by several studies demonstrating that removal of bacteria from *Alexandrium *cultures did not eliminate toxin production (e.g. [18-20]). Along with the lack of conclusive evidence that toxigenesis could be definitively attributed to endosymbiotic bacteria within dinoflagellates, this supports the conclusion that the dinoflagellate genome is responsible for PSP toxin production. As the inheritance of toxin composition follows a Mendelian segregation pattern [21], at least the interconverting enzymes are almost certainly encoded by nuclear genes. Since the early investigations into the biosynthetic pathway of PSP toxins [22], the standing hypothesis has been that this pathway is the same in all PSP toxin-producing organisms, and that the corresponding genes should be homologous. In one of the most-cited papers on his model of the PSP toxin synthesis pathway, Shimizu [23] suggested that the unusual distribution of the ability to produce PSP toxins might be explained by a rare event of horizontal gene transfer from bacteria to dinoflagellates.

A cyanobacterial PSP toxin gene cluster (sxt) has recently been discovered in *Cylindrospermopsis raciborskii *T3 [24]. This cluster contains >35 kb of sequence coding for 26 proteins; PSP toxin synthesis is reported to be initiated with arginine, S-adenosylmethionine and acetate by a new type of polyketide synthase. Similar clusters were found in other PSP toxin-producing cyanobacterial species [25,26], and further studies suggested that at least parts of this cluster have been frequently laterally transferred between different cyanobacterial genera [27]. Nevertheless conclusive evidence of the homology of PSP toxin synthesis in dinoflagellates and prokaryotes is pending.

Attempts to find *Alexandrium *genes associated with PSP toxin production have remained inconclusive. While the physiological and circadian time-frame of PSP toxin production has been elucidated in *A. fundyense *[28], further experiments based on this information have failed to identify candidate genes directly linked to PSP toxins [29]. Subtractive hybridization of cDNA was used to identify toxin-associated genetic differences between toxic and non-toxic subclones of one *Alexandrium tamarense *parental strain [30]. The differential gene fragments, however, did not seem to be directly related to toxins, as shown by polymorphism analysis of other subclones.

Genomic studies in dinoflagellates have long been hampered by their huge genomes, particularly of free-living species - up to 200 pg nuclear DNA cell^-1 ^in *Prorocentrum micans*, which corresponds to roughly 75 times the DNA content of the human genome [31] - and their unusual nuclear composition and organisation (reviewed by [32]). Although most dinoflagellates are functionally haploid in the vegetative stage, the nucleus may contain up to 270 chromosomes [32]. The dinoflagellate nucleus typically lacks histones and nucleosomes and instead contains low abundances of basic histone-like proteins. The chromosomes are permanently condensed, and up to 70% of the nucleotides contain modified or rare bases. This unusual organisation was further illustrated in a low-redundancy sequence survey of the *Heterocapsa triquetra *genome [33]; about 90% of the examined sequences were apparently random, non-repetitive DNA, whereas the highest number of recognizable sequences consisted of repeats, transposons or virus-specific protein sequences. These features pose major difficulties in directly studying the gene content of dinoflagellates, and while some sequencing initiatives are underway, no completely sequenced genome is yet available.

Most of the complexities associated with examining the dinoflagellate genomes can be circumvented by conducting genomic studies at the transcriptomic level [34]. In the last few years, a number of expressed sequence tag (EST) studies has been published (e.g. [35-40]), many of which led to further microarray-based studies of gene expression (e.g. [41,42]). In dinoflagellate species producing polyketide toxins of the spirolide and brevetoxin groups, search strategies based on EST- or cDNA-libraries were successful in identifying a range of polyketide synthases (PKS) [37,43]. These studies made use of the high degree of sequence conservation in several PKS domains (reviewed in [44]), but which of these genes are responsible for production of the corresponding toxins has not yet been established.

As well as being a member of the most well-studied dinoflagellate genus of PSP toxin-producers, for which a wealth of physiological and biosynthetic information on toxin production is available, *A. minutum *is an appropriate model for toxin production studies because of its simple toxin profile. Most *A. minutum *strains produce mainly or exclusively gonyautoxins and *ab initio *both toxic and non-toxic strains are available for comparative studies ([14,45]). We constructed a normalised EST library of *A. minutum *to search for PSP toxin-related genes in this dinoflagellate. To search for toxin-related or toxicity-influencing genes not readily apparent from EST library annotation, we used a microarray approach to compare the transcriptomes of toxic and non-toxic *A. minutum *clones and thus identified differences in gene expression potentially linked to toxin synthesis and/or regulation.

## Methods

### General culture conditions for *Alexandrium minutum*

Unless noted differently, *A. minutum *cultures (origin: Gulf of Trieste, Italy) were grown at 20°C in modified K-medium [46] containing 440 μmol L^-1 ^NO_3_^-^, 36 μmol L^-1 ^NH_4_^+^, 25 μmol L^-1 ^PO_4_^2-^, 10 nmol L^-1 ^SeO_3_^2-^, 1000 μmol L^-1 ^Trizma-Base (pH 8.3), K trace-metal solution and f/2 vitamin solution [47,48]. Illumination was provided from daylight fluorescent lamps at a photon flux density (PFD) of 200 μmol m^-2 ^s^-1 ^on a 16:8-hour light:dark photocycle. All experimental (but not stock) cultures were grown under antibiotic treatment (50 μg mL^-1 ^ampicillin, 33 μg mL^-1 ^gentamicin, 10 μg mL^-1 ^ciprofloxacin, 1.13 μg mL^-1 ^chloramphenicol and 0.025 μg mL^-1 ^streptomycin sulphate) using sterile handling techniques to minimize bacterial influence. To avoid any bias introduced by the antibiotics, treatment was stopped at inoculation of the cultures for toxin content and gene expression experiments, but aseptic handling techniques were maintained.

### Culture and harvest of *A. minutum *for EST library construction

*A*. *minutum *clone AL3T was grown in 800 mL culture bottles for EST library construction. In order to include genes expressed under different physiological conditions, several alternative treatments were applied. Standard condition cultures were grown as detailed above. Two standard condition cultures were subjected to shock treatments (complete darkness or 5°C) for 24 h prior to harvesting. Cultures treated in high salinity (medium supplemented with 15 g L^-1 ^NaCl) and low salinity (medium prepared with 1/3 aged seawater and 2/3 deionised water) were preconditioned in these media for ca. 40 cell cycles (about 40 days, over several sub-culturing transfers) before inoculation of the final cultures. To achieve N-limitation at a reasonably high cell concentration, NO_3_^-^, but not 36 μmol L^-1 ^NH_4_^+^, was omitted from the culture medium. For P-limitation, cultures were grown in medium to which no phosphate was added. Light limitation of growth was attained under a PFD of 60 μmol m^-2 ^s^-1^, and for high and low temperature conditions, cultures were grown at 31.5°C and 13°C, respectively. The cultures were harvested at 7 time points through the photocycle to capture genes expressed at specific time points, and at different stages of the growth curve.

Cells were harvested by filtration upon an 8 μm pore-sized filters and rinsed with filter-sterilized seawater. Cells were immediately transferred into TriReagent (Sigma-Aldrich, Steinheim, Germany), or filters were quick-frozen in liquid nitrogen for later processing.

### EST library construction and automated annotation

RNA was extracted using the Sigma TriReagent protocol, following cell lysis by 10 min incubation at 60°C in TriReagent, aided by repeated vortexing with glass beads included in the sample tube. The resulting RNA pellet was dissolved in 100 μL RNAse-free water (Qiagen, Hilden, Germany). RNA cleanup including on-column DNA digestion (27.3 u DNase per sample) followed the protocol supplied with the Qiagen RNeasy kit. RNA was eluted with 40 μL RNase-free water (Qiagen). When necessary, an additional cleanup and concentration step was applied using Qiagen MinElute or Microcon Ultracel YM-30 columns. RNA purity and quantity were determined with a NanoDrop spectrophotometer (PeqLab, Erlangen, Germany), and integrity and absence of DNA contamination was assessed with a Bioanalyzer (Agilent Technologies, Böblingen, Germany).

Total RNA from harvested cells was pooled as equal amounts from each treatment for construction of a normalised cDNA library. The library was constructed and transformed into electrocompetent *Escherichia coli *cells by Vertis Biotechnologie AG (Freising-Weihenstephan, Germany).

Colonies were picked, and the DNA was extracted by magnetic beads on a robot platform (Qiagen, Hilden, Germany). Plasmid inserts were sequenced from both sides using Big Dye Chemistry (Applied Biosystems, Darmstadt, Germany) and separated on an ABI Prism 3700 × l sequencing (Applied Biosystems) platform. Resulting high quality sequence reads (15,703) were clustered with a sequential assembly using decreasing identity thresholds (gap assembler) to avoid misassemblies due to polyA tails. Contigs were searched for potential alternative splicing by checking alignments of reads in clusters for the presence of gaps in at least one read compared to the consensus sequence of the cluster. Alternative splicing was assumed if the following criteria were fulfilled: 1) the manually inspected difference between the transcripts was at least two bases, and 2) no other polymorphisms were present between the alternative transcripts, thereby indicating transcription from the same locus.

Contig sequences were loaded into SAMS (Sequence Analysis and Management System, Center for Biotechnology, Bielefeld University) for automated annotation based on BLAST comparisons against KEGG, KOG, SwissProt, InterPro and the Genbank nt and nr databases. Sequences were subject to open reading frame (ORF) prediction according to [49], and predicted ORFs were submitted to Pfam, TMHMM-2.0 and SignalP 3.0. To further assist manual annotation, all contigs were analyzed for phylogenetic association to SwissProt sequences using PhyloGena [50]. Annotations of 192 sequences identified from the microarray results as differentially expressed in toxic and non-toxic strains were checked manually.

### In-silico search for known sxt-related genes

The assembled *A. minutum *EST library was screened for contigs showing similarities to any of the publicly available (as of 13.1.2009) sxt-related cyanobacterial sequences in the NCBI protein database using translated protein - nucleotide (blastx) BLAST [51]. Contigs and cyanobacterial sequences producing significant hits (e-value < 0.001) were subjected to a PhyloGena analysis in "top10select" mode to identify the most similar SwissProt sequences and to obtain the most likely protein translation of the EST contig sequence. BLAST-identified pairs of sxt proteins and translated *A. minutum *contigs were combined with the full-length versions of their respective SwissProt hits, and, in the cases of Amin_93i12r and Amin_73a05f, additionally with the 20 most similar nr sequences as identified by BLAST. These sequence sets were used for phylogenetic analysis following the method detailed in [44] with modifications. Briefly, alignments were generated from kalign, and alignment blocks out of the span of both the *A. minutum *and the sxt-related sequence were manually deleted with the CLC sequence viewer version 6.0.1 [52]. Phylogenetic trees were calculated with Phyml_3.0.1 [53] using the subtree pruning and regrafting (SPR) method with a BioNJ starting tree, and the LG model of amino-acid substitution [54] with gamma distribution parameters estimated from the data with four discretised substitution rate classes, the middle of which was estimated as the median. The same settings were used for a 100-replicate Bootstrap analysis.

### Microarray design

Oligonucleotide probes covering both forward and reverse reading directions were designed in collaboration with CeBiTec, Bielefeld University. After a first test hybridisation of a microarray containing 22,264 oligos (2-14 per contig, mean 5.2), the probe set was reduced to the 8,609 best-performing oligos. Following a test for congruence with the contig ORF direction as determined by either manual annotation or a combination of PolyA-Tail identification and the direction of the best SwissProt hit, the best probe was chosen according to performance in triplicate hybridization with standard-strain (AL3T) RNA from 9 different physiological conditions. For some contigs without available manual annotation which could not be automatically assigned an ORF direction, two probes targeting both possible mRNA orientations were retained; these are marked as correct or false during manual annotation.

### Toxin analysis

PSP toxins were extracted following a previously described method [6]. After centrifugation (3,000 × g, 4°C), pellets were suspended in 0.03 N acetic acid and homogenized in FastPrep tubes (Thermo Savant, Illkirch, France) containing 0.9 g of lysing matrix D by reciprocal shaking in a Bio101 FastPrep instrument (Thermo Savant) at maximum speed (6.5 m s^-1^) for 45 s. Cell debris was removed by centrifugation at 16,100 × g at 4°C for 15 min. The supernatant (400 μL) was filtered through a 0.45 mm pore-size spin-filter (Millipore Ultrafree, Eschborn, Germany) by centrifugation for 30 s at 800 × g.

#### Analytical Reagents

Water was deionised and purified (Milli-Q, Millipore, Eschborn, Germany) to 18 MΩ cm^-1 ^quality or better. Formic acid (90%, p.a.), acetic acid (p.a.) and ammonium formate (p.a.) were purchased from Merck (Darmstadt, Germany), nitric acid (p.a.) and phosphoric acid (p.a.) were from AppliChem (Darmstadt, Germany), periodic acid, 1-heptanesulphonic acid, 1-octanesulphonic acid and diammonium hydrogenphosphate were from Sigma (Deisenhofen, Germany). The solvents, methanol, tetrahydrofurane (THF) and acetonitrile, were high performance liquid chromatography (HPLC) grade (Merck, Darmstadt, Germany).

Standard solutions of PSP toxins (saxitoxin, STX; neosaxitoxin, NEO; decarbamoyl saxitoxin, dcSTX; gonyautoxins 1&4, GTX1/GTX4; gonyautoxins 2&3 GTX2/GTX3; decarbamoyl gonyautoxins 2&3, dcGTX2/dcGTX3; and B1) were purchased from the Certified Reference Material Programme of the Institute for Marine Biosciences, National Research Council, Halifax, NS, Canada.

#### Analytical Methods

##### Liquid Chromatography with Fluorescence Detection (LC-FD)

The LC-FD analysis was carried out as previously described in detail [6]. Briefly, PSP toxins were separated by ion-pair chromatography on a ODS reverse-phase analytical column and detected fluorometrically after post-column derivatization. PSP toxins were resolved with two eluants. The first eluant for gonyautoxins and N-sulfocarbamoyl toxins consisted of 6 mM 1-octanesulphonic acid and 6 mM 1-heptanesulfonic acid in 40 mM ammonium phosphate, adjusted to pH 7.0 with dilute phosphoric acid. Components of the saxitoxin group were separated by 0.75% THF and 13 mM 1-octanesulphonic acid in 50 mM phosphoric acid, adjusted to pH 6.9 with ammonium hydroxide, and 15% (v/v) acetonitrile and 1.5% THF. The flow rate was 1 mL min^-1^with the following gradient: initial condition: 100% eluant A and isocratic elution until 15 min, gradient elution to 100% eluant B until 16 min, followed by isocratic elution with 100% eluant B until 35 min.

Post-column derivatization was performed with 10 mM of periodic acid in 550 mM ammonium hydroxide at a flow rate of 0.4 mL min^-1 ^in a reaction coil set at 50°C. Subsequently, the eluate was continuously acidified with 0.75 N nitric acid at a flow rate of 0.4 mL min^-1^. Toxins were detected by a dual monochromator fluorescence detector (λex 333 nm; λem 395 nm). PSP toxin concentrations were determined by external calibration.

##### Liquid Chromatography Coupled with Tandem Mass Spectrometry (LC-MS/MS)

In order to confirm the LC-FD results using an independent method, mass spectral experiments were performed on an ABI-SCIEX-4000 Q Trap (Applied Biosystems, Darmstadt, Germany), triple quadrupole mass spectrometer equipped with a TurboSpray^® ^interface coupled to an Agilent (Waldbronn, Germany) model 1100 LC. The LC equipment included a solvent reservoir, on-line degasser (G1379A), binary pump (G1311A), refrigerated autosampler (G1329A/G1330B), and temperature-controlled column oven (G1316A).

Toxin separation (5 μL of sample injected) for mass spectrometric analyses was performed by a hydrophilic interaction liquid ion-chromatography (HILIC) method [55] with slight modifications. The analytical column (150 × 4.6 mm) was packed with 5 μm ZIC-HILIC, 200 Å, (SeQuant, Haltern, Germany) and maintained at 35°C. A pre-column with the same packing material was also used. The flow rate was 0.7 mL min^-1 ^and gradient elution was performed with two eluants. Eluant A was 2 mM formic acid and 5 mM ammonium formate in 20% water and 80% acetonitrile; eluant B was 10 mM formic acid and 10 mM ammonium formate in water. The gradient was as follows: 20 min column equilibration with 20% eluant B, linear gradient to 35% B until 5 min, then linear gradient to 40% B until 10 min, then linear gradient to 45% eluant B until 20 min, followed by isocratic elution with 45% eluant B until 24 min and finally return to initial 20% eluant B until 25 min.

Multiple reaction monitoring (MRM) experiments were carried out in positive ion mode by selecting the following transitions (precursor ion > fragment ion): *m/z *412 > 332 and *m/z *412> 314 (for GTX1/GTX4 and C3/C4), *m/z *396> 316 and *m/z *396> 298 (for GTX2/GTX3, C1/C2 and B2), *m/z *380> 300 and *m/z *380> 282 (for B1), *m/z *353> 273 (for dcGTX2/dcGTX3), *m/z *369> 289 (for dcGTX1/dcGTX4), *m/z *300> 282 and *m/z *300> 204 (for STX), *m/z *316> 298 and *m/z *316>196 (for NEO), *m/z *257>196 and *m/z *257> 156 (for dcSTX) and *m/z *273> 255 (for dcNEO). Dwell times of 50-150 ms were used for each transition with the following source parameters: curtain gas: 30 psi, temperature: 650°C, ion-spray voltage: 5000 V, gas 1 and 2: 70 psi, interface heater: on, collision gas: high, declustering potential: 66 V, entrance potential 10 V, collision energy: 30 V, and collision cell exit potential: 12 V.

### Determination of harvesting time for gene expression analysis

To determine the optimal point of the light-dark cycle at which putative toxin genes are most likely to be expressed, triplicate *A. minutum *AL3T cultures were grown in 5 L glass flasks with constant gentle aeration under controlled standard conditions, as previously described. Cultures were sampled for cell counts and toxin analysis at the onset of dark phase, at the onset of light phase, and at 2-h intervals until the beginning of the next dark phase, with a sterile tube-sampling system [56,57]. Duplicate samples for toxin analysis (125 - 200 mL of culture depending on harvesting time) were harvested by centrifugation for toxin analysis by LC-FD.

### Inter-clonal comparisons of gene expression

Gene expression was compared between toxigenic (AL3T and AL9T) and naturally non-toxic (AL1T) *A. minutum *clonal strains originating from the same geographical population in the Gulf of Trieste, Italy (isolated by A. Beran; see [58]). Bacteria-reduced triplicate cultures were grown under the control conditions as stated above. Culture growth was monitored by daily manual cell counts of samples fixed in Lugol's iodine solution.

The toxin content of duplicate samples containing at least 2 × 10^6 ^AL3T and AL9T cells and non-duplicate samples containing at least 4 × 10^6 ^AL1T cells were analyzed by LC-FD. To confirm the toxin identification of specific PSP toxin analogues by LC-MS, samples of additional parallel cultures were combined into pellets containing 8.5 × 10^6 ^to 1.1 × 10^7 ^cells.

Samples for RNA extraction were taken during exponential growth phase, at 10-11 h (Sampling Time 1, ST1) and at 6-7 h (Sampling Time 2, ST2) after onset of light phase. Total RNA (500 ng sample^-1^) was extracted as described herein, then amplified and labelled with a Low RNA Input Linear Amplification kit (Agilent, Waldbronn, Germany). The Agilent Low RNA Input Linear Amplification Kit protocol was followed for synthesis of Cy3- and Cy5-labelled cRNA and microarray hybridisation. Microarrays were scanned on an Agilent G2565AA scanner, and raw data were extracted with the Agilent Feature Extraction Software version 9.1.3.1 (FE). Array quality was monitored with the Agilent QC Tool (v1.0) with the metric set GE2_QCMT_Feb07.

Pre-processed data were subjected to SAM (Significance Analysis of Microarrays [59]) as implemented in MeV 4.0 [60], and SAM-based q-values [61] were calculated. Probes with a q-value of <1% were considered to indicate differential expression of the corresponding genes if the mean fold change of the sample triplicate was at least 1.5. To minimise the influence of physiological differences between the strains that were not related to the capacity for toxin production, only genes identified as significantly higher or significantly less expressed in both toxic strains at both sampling time-points were designated as "higher expressed" or "less expressed" in toxic strains.

Samples were clustered by Hierarchical Cluster Analysis (HCL) support trees as implemented in MeV 4.2. Trees calculated with different distance measures (Euclidean, Manhattan and Covariance values) and different distance metrics (average, complete and single linkage) were compared. Node confidence was tested by 1000 Bootstrap replicates.

To test reliability of the microarray, expression levels of six genes were evaluated by qPCR. Four of these genes had been identified as significantly higher expressed in the toxic strains, the other two as significantly higher expressed in the non-toxic strain. The qPCR was carried out as described [62] with the lepidopteran genes MA and NSP as an artificial internal reference. Primers for qPCR were designed with PrimerExpress 3.0 (Applied Biosystems, Darmstadt, Germany) and synthesized by Eurofins MWG Operon (Ebersberg, Germany). Standard curves using cDNA plasmids corresponding to target sequence ESTs were plotted to test the primer pairs for consistent efficiency at different concentrations. These plasmids were amplified by M13-primed PCR; qPCR primers were tested on dilution series of plasmid PCR products spanning at least 8 orders of magnitude. The qPCR reaction was based on the PowerSybrGreen PCR Master Mix (Applied Biosystems) according to the manufacturer's instructions, using a 7000 Real-Time PCR System (Applied Biosystems).

Prior to cDNA synthesis the samples were spiked with the artificial internal control RNAs MA (1 ng reaction^-1^) and NSP (1 pg reaction^-1^). Equal amounts of RNA from all strains were processed in parallel. All qPCR reactions based upon the same primer-set were run on the same plate, and reaction efficiencies were compared with MA and NSP-specific primers. To test for contamination with genomic DNA, negative controls consisted of full reactions in which cDNA was exchanged for RNA aliquots of all samples. Thresholds were determined manually for each primer set. Relative expression levels were recorded as the cycle threshold value (C_t_).

### PCR

To test for the presence of the cyanobacterial toxin gene cluster in *A. minutum*, amplification of the cyanobacterial genes from genomic DNA isolated from AL3T cultures grown under antibiotic treatment was attempted. The 14 primer pairs designed against the PSP toxin ORF in the *Raphidiopsis brookii *genome (K. Stucken et al., unpublished data; Additional file 1) were tested. A primer pair targeting the D1/D2 [63] region of the large ribosomal subunit was used as positive control, primers against the bacterial 16S ribosomal subunit controlled for bacterial contamination of the DNA.

Reactions were scaled to 30 μL and contained 20 ng genomic DNA, 0.2 mM forward and reverse primers, 0.1 mM dNTPs, 0.6 units of U of HotMaster *Taq *DNA polymerase (Eppendorf) and HotMasterTaq buffer 1× (Eppendorf). Gradient PCR was employed to try each primer at 6 different annealing temperatures spanning a range of approximately 9°C. As the primers were of different melting temperatures, three different temperature spans between 46 and 58°C were used. Cycling conditions were based on those optimal for these primers (Additional file 1): initial denaturation at 94°C for 5 min, 20 cycles with denaturation 94°C (20 s), annealing for 45 s, extension at 70°C (30 s), and a final extension step of 10 min at 70°C. PCR products were visualized after agarose gel electrophoresis.

## Results

### Characterisation of the EST Library

The cDNA fragments sequenced from both sides yielded 15,703 read sequences clustered into 4,320 contigs. Of these contigs, 3,112 (72%) combined the forward- and reverse reads of at least one bacterial clone, and thus can be considered full-length insert sequences, but might not represent the total length of the corresponding mRNA. Among contigs, 1,998 contained reads from more than one cDNA insert. Contig length varied between 100 and 2185 bp, with a mean of 791 ± 240 bp (mean ± standard deviation). The mean GC content of contigs was 56.10 ± 4.67% (Table 1).

**Table 1:** Base composition of the *A. minutum *EST library

GC content	no. of contigs	fraction of contigs
**<40%**	75	1.7%
**40-50%**	243	5.6%
**50-60%**	3454	80.0%
**≥ 60%**	548	12.7%

**22%**	minimum	
**66%**	maximum	

The examination of alignments of reads in the clusters for potential alternative splicing identified different splice variants in ca. 9% of the contigs containing reads from more than 1 cDNA insert, or ca. 3% of all genes examined.

Checking all contigs for evidence of transsplicing revealed that the complete dinoflagellate-specific spliced leader sequence (SL), DCCGTAGCCATTTTGGCTCAAG (D = U, A, or G) [64,65] was present in 204 contigs. Of these SLs, 37 (18%) started with A, 10 (5%) with G and 157 (77%) with U. In addition, we identified 35 contigs containing incomplete SL sequences. In 23 of these, the sequence was lacking 1-4 nucleotides at the 5'-end of the contig, while 23 SLs differed in 1-3 nucleotides within the SL sequence. Of the SL-containing contigs, 193 contained an additional 5'polyA-region and thus are likely to contain the complete ORF (see Additional file 2).

**Figure 1:**
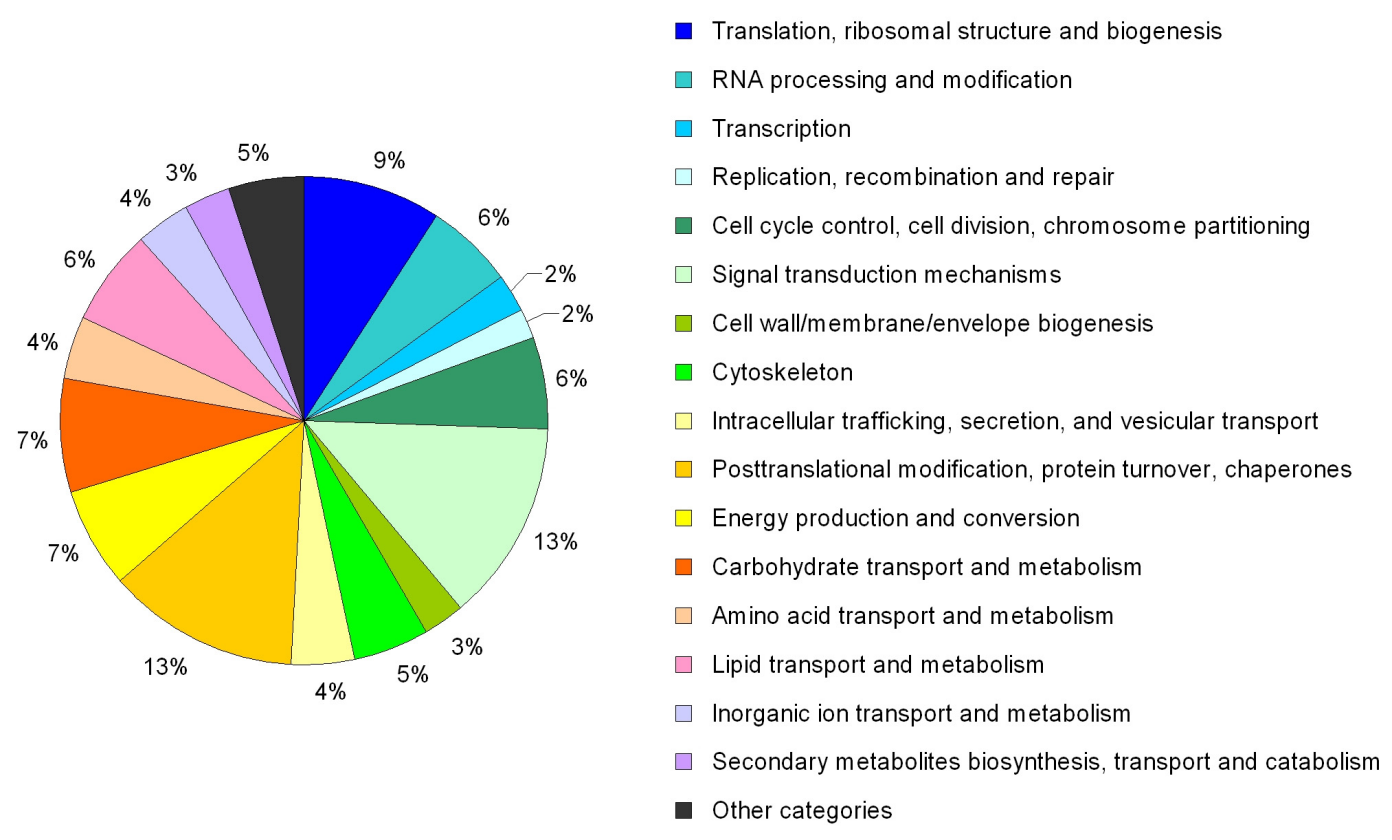
**KOG-based functional classification of 1005 contigs as obtained by BLAST-identified similarities**.

Automated annotation with the SAMS platform yielded annotations for 1,200 contigs (28%), whereas 1,969 contigs (46%) did not produce any BLAST hit below tool cut-off. Another 1,151 contigs (27%) produced no hits that met the criteria for automated annotation. Automated classification to KOG categories based on the SAMS "BLAST 2× *vs*. KOG" output (cut-off e-value 10^-4^) resulted in assignment of 1005 contigs (23%) to functions other than "General function prediction only" or "Function unknown" (Fig. 1).

A BLAST survey of the *A. minutum *library against all available dinoflagellate EST libraries available in GenBank (as of 28.11.2007) plus a new *A. ostenfeldii *EST library (N. Jaeckisch, unpublished observations) detected similar sequences for 43% of the contigs at a moderately stringent cut-off value of e = 1 × 10^-10 ^(Table 2).

**Table 2:** Result of BLAST search of *A. minutum *contigs against all dinoflagellate ESTs available in GenBank (as of 28.11.2007).

E-value cut-off	all available dinoflagellates		*A. tamarense*		*A. catenella*		*A. ostenfeldii*	
**10^-5^**	2209	(51.1%)	1487	(34.4%)	940	(21.8%)	1543	(35.7%)
**10^-10^**	1864	(43.1%)	1203	(27.8%)	715	(16.6%)	1335	(30.9%)
**10^-30^**	1123	(26.0%)	703	(16.3%)	360	(8.3%)	867	(20.1%)

### In-silico search for sequences related to the cyanobacterial PSP toxin gene cluster

A BLAST search of the EST library against the cyanobacterial PSP toxin genes resulted in 14 hits at e-value <0.001. In 12 of 14 maximum likelihood phylogenies calculated from the BLAST-identified pairs of sxt proteins and translated *A. minutum *contigs, and their closest matches identified from the SwissProt database, the *A. minutum *and the cyanobacterial sequences clustered in different clades. Furthermore, these clades were separated by long branches, well-supported nodes with high bootstrap support, or both (see Additional file 2: Phyml-based likelihood trees with bootstrap support values). In the case of Amin_93i12r and Amin_73a05f, the phylogenies based on SwissProt hits were not that clear (Additional file 2 M.1 and N.1). This led to a further test for orthology of Amin_93i12r and Amin_73a05f with the cyanobacterial PSP toxin genes by including the results of a BLAST search against the nr database. In the case of Amin_93i12r, the cyanobacterial gene in question was identified in this BLAST search as hit number 64; the BLAST result list of Amin_73a05f did not include its potential cyanobacterial counterpart within the best 100 hits. When we repeated calculation of the phylogenies for Amin_93i12r and Amin_73a05f with the best 20 BLAST *vs*. nr hits included in addition to the SwissProt database hits, the *Alexandrium *sequences did not cluster in the vicinity of the cyanobacterial PSP toxin genes (Additional file 2 M.2 and N.2).

### Toxin production over the light-dark cycle

**Figure 2:**
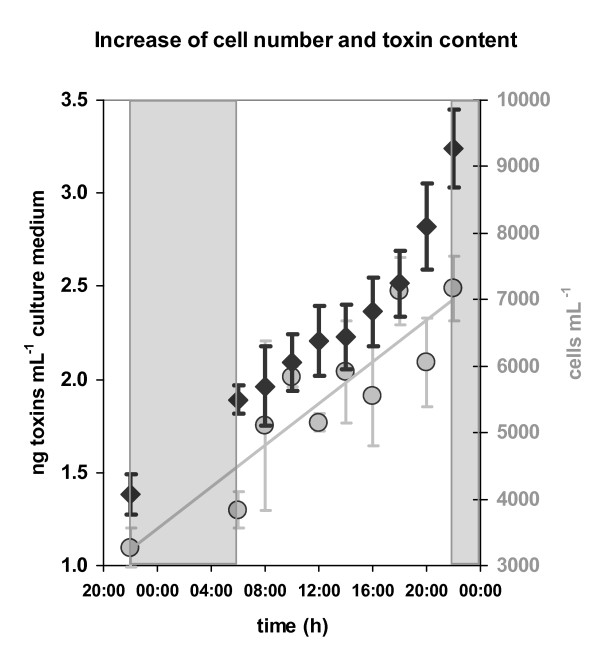
**Increase of cell number and culture toxin content in strain AL3T over 24 hours**. Grey boxes = dark phase, circles = cell numbers, diamonds = total PSP toxins mL^-1^.

Under our standard growth conditions, the toxin-producing strain AL3T exhibited a growth rate (1.01 cell divisions day^-1^; I. Yang, unpublished data) that approximated the total length of the light:dark period, which suggested that these cultures might have a naturally circadian-phased cell cycle. In the triplicate cultures examined, however, both cell number and culture toxin content increased over the entire light phase. The toxin content per cell stayed roughly the same during the whole day, while cell numbers doubled from 3,271 ± 296 to 7,167 ± 482 cells mL^-1 ^within 24 h. The toxin content of the culture increased from 1.38 ± 0.16 ng μL^-1 ^to 3.24 ± 0.21 ng μL^-1 ^(Fig. 2)

### Strain-specific toxin content

**Figure 3:**
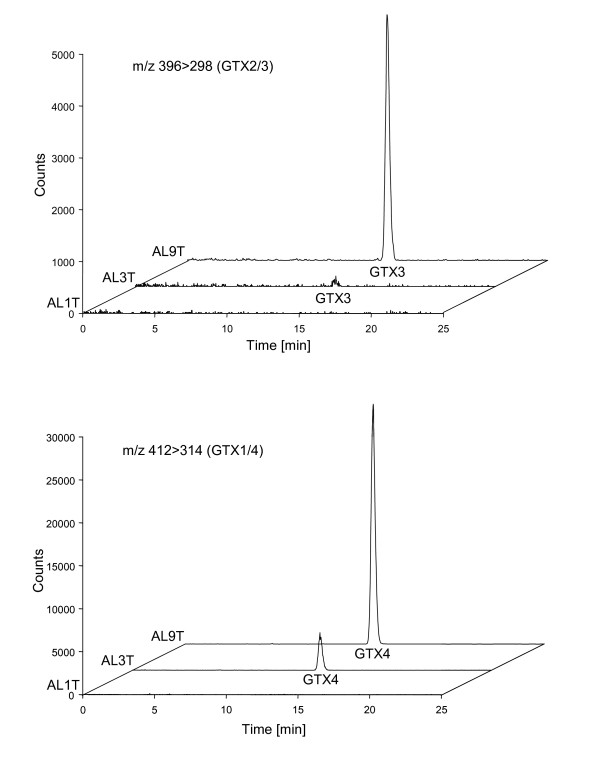
**Extracted ion traces of LC-MS/MS chromatograms of the *A. minutum *strains AL1T, AL3T and AL9T**. Top: transition 396 > 288 for GTX2/3 (retention time GTX3: 14.1 min); bottom: transition 412 > 314 for GTX1/4 (retention time GTX4 13.3 min)

*A. minutum *PSP toxin concentration as measured by LC-FD was strongly clone-dependent. No PSP toxins were found in AL1T, whereas the toxin profiles of AL3T and AL9T were virtually identical, although toxin cell quotas differed by an order of magnitude (Table 3). The toxin profiles as confirmed by LC-MS/MS (Fig. 3) in the extracted ion chromatograms clearly indicate the presence of the transitions *m/z *396 > 298 for GTX3 and *m/z *412 > 314 for GTX4. No signals were obtained from any other ion traces, indicating that no known PSP toxins other than GTX3 and GTX4 were present in the samples.

**Table 3:** Strain-specific toxin content as measured by LC-FD (in fmol cell^-1 ^± standard deviation, n = 3).

strain	Aggregate toxins	GTX 1/4	GTX 2/3	STX
**AL3T**	1.11 ± 0.06	1.09 ± 0.06	0.02 ± 0.003	traces
**AL9T**	10.33 ± 1.95	10.02 ± 1.94	0.29 ± 0.01	0.02 ± 0.001
**AL1T**	0	0	0	0

### Gene expression

Gene expression differences between toxic and non-toxic isolates were identified by microarray experiments comparing the two toxic strains AL9T and AL3T to the non-toxic AL1T. With SAM at a gene-specific FDR cut-off of q = 1% and a fold-change cut-off of 1.5, 145 contigs were identified as higher expressed in both toxic strains at both time points tested, whereas 47 genes were significantly higher expressed in the non-toxic strain (see Additional file 3 - List of differentially expressed genes). HCL support trees in which samples were clustered according to similarity of gene expression grouped the samples according to their clonal designations. Of the nine tested different combinations of clustering parameters, only the tree based on the Euclidean distance measure combined with complete linkage separated the midday and afternoon triplicates of the AL9T vs. AL1T microarrays. In all other analysis, samples did not cluster according to harvesting time.

Of the 145 genes identified as higher expressed in the toxic strains at both time points, 49 could be manually annotated based on BLAST-based automated annotation, domain comparison using Pfam and phylogenetic reconstruction with the PhyloGena program [50]. Putative functions could be assigned to 31 of these genes (Table 4).

**Table 4:** Putative functions of differentially expressed genes

Contig name	Gene product	Putative function	Log mean fold changes		
			AL3T/AL1T	AL9T/AL1T	Tox/Nontox
**Higher expressed in toxic strains**					
Amin_34h03r	putative small nuclear ribonucleoprotein polypeptide E	gene expression	1.7	2.4	2.0
Amin_36k19f	translation elongation factor-like protein	gene expression	24.4	25.8	25.1
Amin_44h09f	glycyl-tRNA synthetase	gene expression	4.3	4.6	4.5
Amin_56k21r	probable translation initiation factor E4	gene expression	22.6	28.5	25.4
Amin_65d02r	putative Ubiquitin-like domain-containing CTD phosphatase 1	gene expression	3.1	2.3	2.7
Amin_75e04r	putative helicase	gene expression	24.3	26.1	25.2
Amin_48i15f	putative helicase	gene expression	26.9	44.8	34.7
Amin_56g23f	putative Alpha-L-fucosidase 1	protein glycosylation	1.8	5.5	3.2
Amin_95c08r	sialyltransferase	protein glycosylation	20.3	23.5	21.9
Amin_87p16r	casein family protein kinase	signal transduction	9.7	13.0	11.2
Amin_98a08r	endonuclease/exonuclease/phosphatase family protein	intracellular signalling	3.6	4.8	4.2
Amin_63e03r	hypothetical protein similar to Sentrin-specific protease 8	cell cycle control	3.7	3.2	3.4
Amin_68d07f	rrm family protein similar to mei2	cell cycle control	2.9	2.5	2.7
Amin_63c06r	RNA-binding protein similar to mei2	cell cycle control	1.9	2.6	2.2
Amin_53e04r	chlorophyll a-c binding protein	chloroplast	5.5	4.9	5.2
Amin_81i24r	translation elongation factor P	organellar translation	37.4	45.2	41.1
Amin_97a05r	putative mitochondrial import receptor subunit tom40	mitochondrial	4.6	5.8	5.1
Amin_70g03r	ferrochelatase	mitochondrial	3.0	2.8	2.9
Amin_40e21r	putative hydrolase	hydrolytic enzyme	5.5	3.1	4.1
Amin_82n24r	phospholipase/carboxylesterase family protein	hydrolytic enzyme	7.2	8.8	8.0
Amin_46a02r	Abhydrolase domain-containing protein	hydrolytic enzyme	5.6	4.0	4.7
Amin_95b07r	putative sulfatase, similar to Ats family arylsulfatases	hydrolytic enzyme	3.2	4.2	3.7
Amin_79g11r	hypothetical protein similar to various hydrolytic enzymes	hydrolytic enzyme	2.1	3.4	2.7
Amin_62h07r	hypothetical protein similar to glutathione S-transferase	detoxification	4.7	7.0	5.8
Amin_12c01r	aminotransferase, class I or II	Amino-transferase	2.1	3.0	2.5
Amin_93i12r	hypothetical protein similar to branched-chain-amino-acid aminotransferase	Amino-transferase	2.5	4.2	3.3
Amin_46c04r	hypothetical protein similar to Iron/ascorbate family oxidoreductases	Oxido-reductase	7.9	8.8	8.3
Amin_83a03f2	galactose-1-phosphate uridylyltransferase	sugar metabolism	5.2	8.6	6.7
Amin_44a04r	putative steroid oxidoreductase superfamily member	secondary metabolites	3.2	4.0	3.6
Amin_53f01f	putative lipolytic enzyme, G-D-S-L family	lipid metabolism	3.5	2.3	2.8
Amin_17d04f	putative CorA-like Mg2+ transporter protein	metal ion transport	3.5	3.7	3.6
Amin_59d02r	GFA family protein	putative carbon-sulphur lyase	20.4	22.8	21.6
					
**Higher expressed in non-toxic strain AL1T**					
Amin_30a05r	cyclin-dependent kinases regulatory subunit 1	signal transduction	-3.9	-2.9	-3.3
Amin_74g08f	dynein heavy chain family protein	intracellular transport	-5.5	-4.3	-4.9
Amin_44h03r	fibronectin type III domain-containing protein	putatively involved in signalling	-9.2	-11.3	-10.2
Amin_09b02f	glutaredoxin family protein	DNA nucleotide synthesis	-4.7	-3.2	-3.9
Amin_89h11r	hypothetical protein containing a putative 'Cold-shock' DNA-binding domain	stress response	-2.4	-3.2	-2.8
Amin_101h04r	inorganic H+ pyrophosphatase, 3'-region.	proton pump	-3.0	-1.9	-2.4
Amin_16h04r	peptide chain release factor 1	translation	-8.5	-7.5	-8.0
Amin_42k04f	hypothetical protein similar to taurine catabolism dioxygenase TauD	putative dioxygenase	-2.7	-29.7	-9.0

The group of 47 genes higher expressed in the non-toxic strain AL1T contained 8 genes for which a putative function could be identified (Table 4).

For 8 of the contigs higher expressed in the toxic strains, the signals of the non-toxic strain were identified as not "well above background" by the microarray data extraction software on at least 8 of the 12 arrays, and on at least one array for each strain-time-point combination. We consider these as either not expressed in the non-toxic strain AL1T or considerably different on the sequence level, which might imply functional differences. This group contains two putative helicases and six hypothetical proteins for which no annotation could be found. For one of these, Amin_66c10r, we found a homologous sequence in the published *A. tamarense *library [35], but in none of the libraries of non-PSP toxin-producing dinoflagellates. Another five sequences examined did not produce significant BLAST hits against any other dinoflagellate library (see Additional file 3).

**Figure 4:**
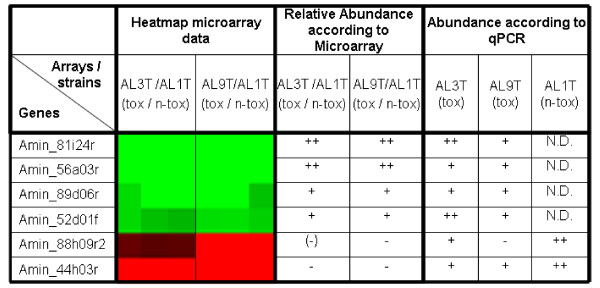
**Comparison of expression ratios based on microarray- and qPCR- data**. Array data: more than 15× higher expressed in toxic strains, ++; more than 5×, +; more than 5× higher expressed in non-toxic strain, -; 2-5× higher expressed in non-toxic strain, (-); raw data see supplementary table 1. qPCR: Ct < 25, ++; Ct<27, +; Ct>27, -; N.D., not detected at Ct < 35.

The analysis of relative mRNA levels of 6 genes re-examined by qPCR, as shown in Fig. 4, indicated that microarray and qPCR data agreed qualitatively, although most of the fold changes calculated from qPCR results were substantially higher than those identified by microarray.

## Discussion

### EST Library

The EST library presented here includes sequences from a variety of physiological states induced by different treatments and sampling time points. We aimed for a high diversity of transcripts ideally representing an overview over the complete transcriptome of this species, by combining samples from control, cold-shocked, darkness-shocked, high- and low-salinity stressed, nitrogen- and phosphorous-starved as well as heat- and cold-stressed *A. minutum *cultures and by normalization of the cDNA library. The 15,703 read sequences, which originated from 9,485 cDNA clones, clustered into only 4,320 contigs or putative genes. We estimated that our contigs account for approximately 80% of the potential genes within this particular cDNA library, based upon the rarefaction curve analysis (Additional file 4). Nevertheless, we recognize that not 80% of the potential gene candidates from the *A. minutum *genome are included in the library. Based on the experience of diverse genome projects, the total number of gene candidates is probably two or three times higher. In any case, contig numbers for the *A. minutum *library are substantially lower than those found for EST libraries of the congeneric *A. tamarense *(6,723 unique sequences from 11,171 reads, normalised library) [35] and *A. catenella *(6,496 "unigenes" from 10,850 ESTs, library not normalised) [38]. The latter two EST libraries were both obtained from cultures grown only under control conditions, and hence presumably do not reflect stress, cell cycle or different culture stage effects on gene expression. The relationship of unique genes within the EST library for *A. minutum *is reminiscent of that of the free-living marine dinoflagellate *Heterocapsa triquetra *[39], for which the EST library was based on samples taken throughout the photocycle, but contained only 2,022 unique clusters out of 9,309 ESTs.

A BLAST-search of the *A. minutum *contigs against available dinoflagellate EST libraries found at least weakly similar sequences for about half of the contigs, which is mainly due to the availability of EST libraries for *A. tamarense *[35], *A. catenella *[38] and *A. ostenfeldii *(N. Jaeckisch et al, unpublished observations) (Table 2). The low novelty rate implied by these similarities, however, is not reflected in the annotation results; automated annotation was possible for 28% of the contigs. The attribution of these contigs among functional categories (depicted in Fig. 1) is likely to reflect to a large extent the sequence conservation in the respective groups, and not necessarily the true distribution of gene functions. Studies examining the function of dinoflagellate genes are still rare, and as they are ecologically, physiologically and genetically very distinct from their better-studied relatives, the apicomplexa [66], annotation of dinoflagellate genes remains notoriously difficult (see [67]).

A recent study [68] suggests that gene expression in dinoflagellates reflects genomic organisation. Most highly expressed genes seem to be in tandem arrays of slightly different gene copies, with few or no introns, short intergenic spacers, and are mostly spliced-leader trans-spliced [64,65]. We found evidence for the existence of such gene families for 8 of 9 sequences tested; in addition to intra-specific differences among strains, there was considerable intra-strain variability on both the transcriptomic and genomic levels, with variation in the cDNA within a strain, comprising both synonymous and non-synonymous differences. This implies that microarray-measured differences in gene expression might be due to either differences in the frequencies of highly probe-complementary *vs*. less probe-complementary gene family members, or to the simultaneous up- or down-regulation of whole gene families.

In contrast to these multi-copy genes, moderately expressed genes appear to be single copy and exhibit a higher intron density; trans-splicing seems to occur less often [68]. Furthermore, we identified alternative splice variants in 9% of the genes containing reads from more than one cDNA, adding to the evidence that more conventional eukaryotic introns and splicing phenomena exist in dinoflagellates [69]. Based on our data and with reference to previous studies we cannot, however, exclude that in rare cases identical copies with different splicing patterns may exist. Yet since splicing accuracy and efficiency depends on the underlying sequence, this scenario is highly unlikely. In any case, to our knowledge, our work herein is the first report on the frequency of alternative splicing in dinoflagellates.

### In-silico search for candidate saxitoxin-related genes

The known information on PSP toxin-related proteins in dinoflagellates is limited to enzymes involved in the interconversion of PSP toxins, which have been characterised on the protein level. An oxidase capable of transforming GTX 3 to GTX 4 has been found in crude extracts of *A. tamarense *cells [70]. In the dinoflagellate *Gymnodinium catenatum *the same research group also reported an N-sulfotransferase forming C2 from GTX3 in a reaction mix containing adenosine 3'-phosphate 5'-phosphosulfate. Nevertheless, this enzyme was present in both toxic and non-toxic isolates of this species. A similar enzyme was also found in a crude enzyme extract of *A. tamarense *[71]. A sulfotransferase that specifically transfers a sulfonyl residue to a carbamoyl group present in some saxitoxin analogues has been purified and characterised from PSP toxin-producing *G. catenatum *[5]. The authors suggested that STX might be the only toxin directly synthesized, and that all other PSP toxins are derived by a fixed set of interconversions. Consistent with this idea, an O-sulfotransferase specifically transferring a sulfonyl group to O-22 of 11-hydroxy-STX to produce GTX 3 has also been purified and characterized from *G. catenatum *[72]. If these interconversions are the same in all PSP-producing dinoflagellates, *A. minutum *should contain the characterized O-sulfotransferase [72] and the oxidase transforming GTX 3 to GTX 4 [70]. Searching our *A minutum *EST library for corresponding candidate genes revealed several oxidases and one sulfotransferase of unknown specificity. The microarray data set shows two of the potential oxidases as higher expressed in the toxic strains - one hypothetical protein similar to iron/ascorbate family oxidoreductase was between 6.6 and 10.3 times higher expressed in the toxic strains AL3T and AL9T, and a putative steroid oxidoreductase superfamily member 2.9 to 4.5 times. Both oxidases had a very good BLAST match with the PSP toxin-producers *A. tamarense *and *A. catenella*, respectively, but only weak or no similarities to other dinoflagellate sequences. The sulfotransferase had a potentially homologous match (BLAST e-value = 6 × 10^-11^) in the *A. tamarense *EST library, but in none of the other dinoflagellate libraries. The microarray data did not identify it as differentially expressed between toxic and non-toxic strains. As sulfotransferases and oxidases are rather general enzyme types occurring in many other pathways including those of primary metabolism, it is not possible to determine at this point whether one of the enzymes found in *A. minutum *is related to PSP toxin modification.

The only available sequence information related to PSP toxin synthesis is a cyanobacterial gene cluster first identified in *C. raciborskii *[27]. The standing hypothesis is that PSP toxin biosynthesis should follow the same pathways [22] and be encoded by the same set of genes in dinoflagellates and cyanobacteria. This is usually explained by horizontal gene transfer (HGT) between the prokaryotic cyanobacteria and dinoflagellates [23,73,74]. Although the gene cluster coding for the enzymes of the PSP synthesis pathway are known, corresponding genes in dinoflagellates have not been identified to date.

Searching our *A. minutum *EST library for contigs homologous to the cyanobacterial PSP toxin genes identified several sequences of moderate similarity. However, when we calculated phylogenies from these sequence pairs and their most similar matches as identified by BLAST against SwissProt or, in the case of *Amin_93i12r *and *Amin_73a05f *against SwissProt and nr, the *A. minutum *sequence and the *C. raciborskii *toxin gene appeared to be very distant relatives separated by many more closely related sequences and often by long branches in the Phyml-based likelihood trees. This indicates that if a suite of genes similar to the *C. raciborskii *PSP toxin gene cluster is present in the *A. minutum *transcriptome, none of its sequences were recovered during EST library preparation.

A gradient PCR test using DNA from *A. minutum *AL3T cultures grown under antibiotic treatment and primers designed against the PSP toxin cluster from cyanobacteria recovered no PCR products except the positive control (I. Yang, unpublished data). However, negative results in a PCR experiment cannot prove non-existence of a sequence, and other primers or different PCR conditions might lead to a different outcome. In any case, no evidence of the cyanobacterial PSP toxin gene cluster in dinoflagellates has been published, in spite of the fact that the PSP-producing *Alexandrium *species are among the best-studied dinoflagellates (e.g. [35,37,38]). Together with the data presented here, this suggests the hypothesis that the PSP toxin genes in dinoflagellates are rather more different from their cyanobacterial counterparts than would be expected in the case of a recent gene transfer. Whether or not this HGT event has occurred as hypothesized, or if the capacity for PSP toxin production arose independently in dinoflagellates, or if the bacterial genes were horizontally transferred to dinoflagellates but then changed during evolutionarily relevant time-spans to a point beyond recognition with current methods, can only be determined when the first dinoflagellate PSP toxin genes are identified.

### Physiology-based approach

Although it has been suggested [43] that many genes in dinoflagellates are post-transcriptionally regulated, examination of mRNA pools regularly succeed in finding genes differentially expressed between different physiological treatments or at different times of the light-dark cycle [29,41,42,75]. The ability to produce PSP toxins is genetically fixed within strains, and as it is probably genetically determined, evidence should be traceable to the mRNA pool, whether transcriptionally or post-transcriptionally regulated. This interpretation serves to justify our attempt to identify possible candidate genes for involvement in PSP toxin production by comparing mRNA pools of toxic and non-toxic strains.

Certain experimental efforts to understand PSP toxin production in *Alexandrium *species have focussed on identifying critical time-points during the cell cycle. For example, in light-deprivation synchronized cultures of *Alexandrium fundyense*, PSP toxin production was shown to be coupled to an 8-10 h period in the G1 phase of the cell cycle [28]. Several dinoflagellate species are reported to exhibit naturally phased cell cycles in both non-synchronized cultures and in field populations [76-78]. As the toxic *A. minutum *strain AL3T is capable of a growth rate of one cell division per day under optimal conditions, we suspected that even experimentally non-manipulated cultures might exhibit a circadian-phased (perhaps even synchronised) cell cycle similar to that found for the dinoflagellate *Amphidinium operculatum *[79]. However, both cell numbers and intracellular toxin per culture volume increased continuously throughout the light phase (see Fig. 2) in non-synchronised AL3T cultures. A similarly broad time frame for cell division in a dinoflagellate is only known for a slow-growing culture of *Gyrodinium uncatenatum *[80] The apparent lack of phased cell division in non-synchronised *A. minutum *AL3T cultures might be an artefact of growth under conditions optimised for high growth rate and therefore may not reflect growth under natural conditions or in experimentally synchronised cultures. However, the failure of most gene expression - based HCL support trees to resolve the difference between mid-day and afternoon triplicates of the AL3T - AL1T and AL9T - AL1T-comparisons indicate that a similar lack of phased physiological conditions might occur in other *A. minutum *strains grown under the same conditions, although we did not examine the timing of cell division and toxin production in the other strains in this study. Both laboratory cultures and field population of different *Alexandrium *species are known to be primarily in G1 throughout most of the light period or daylight time [28,81,82]. Thus, combining data from two different time-points during light phase should capture differences in the mRNA pool of toxic and non-toxic strains during PSP toxin production while excluding strain-specific differences in circadian responses not related to toxin production.

The microarray approach can identify differences in mRNA abundances of known sequences irrespective of the assigned function, to compare the gene expression of the different strains. This allows screening for gene expression patterns associated with different genetic traits coupled to physiological responses such as toxin production, and can be used to correlate non-annotatable sequences with biological connotation [83]. As the two-colour microarray setup used in this study directly measures the ratio of sequence abundances in the two samples hybridized, the detection of these differences is largely independent of overall expression levels.

In inter-strain comparisons using microarrays, mRNA differences between the strains might result in differences in hybridisation efficiency which from the array data alone cannot be distinguished from expression level differences. However, comparison of microarray results (see Additional file 3:_List of differentially expressed genes) with qPCR data (Fig. 4) indicated that in this respect, our microarray setup is more likely to pick up slightly divergent sequences than the gene expression "gold standard" qPCR.

According to microarray data we identified 145 genes as higher expressed in both toxic strains at both time-points examined. Among the eight of these that were not significantly expressed in the non-toxic strain were two putative helicase sequences and six hypothetical proteins for which no annotation could be found. We consider these eight genes as candidates for genes associated with the biosynthesis or regulation of PSP toxins, or for adaptive responses to intracellular PSP toxins. For one of the non-annotatable candidates we obtained a significant BLAST hit (E-value 6x10^-14^) against ESTs of the PSP toxin-producing species *A. tamarense*, but not against any of the non-PSP-toxin-producing dinoflagellates tested. As we do not know the proportion of the respective transcriptomes represented in either EST library, we rate the match but not the lack of matches as significant. Therefore we consider these eight genes candidates for genes associated with the biosynthesis or regulation of PSP toxicity, or for acclimatisation to intracellular PSP toxins.

The putative functions of 31 of the genes higher expressed in the toxic strains, and of 8 of the 47 genes higher expressed in the non-toxic strain AL1T, were identified by manual annotation. A number of these were involved in post-transcriptional processes, which in dinoflagellates seems to be an important level of gene regulation [68].

Most of the gene expression differences mentioned herein are probably due to variations in growth rate, overall metabolic rate, or other physiological differences between individual strains. Nevertheless, the genes that were not significantly expressed in the non-toxic AL1T match the expression pattern expected for genes involved in toxin biosynthesis, regulation or sequestration. We therefore propose a particular focus on these sequences as gene targets to be further comparatively studied in other dinoflagellates and species complexes for which toxigenic and non-toxigenic strains are available. All authors read and approved the final manuscript.

## Conclusions

An in-silico approach to identify genes related to the recently published cyanobacterial PSP toxin gene cluster in our new EST library of the PSP toxin-producing dinoflagellate *A. minutum *did not yield evidence for cyanobacteria-like sxt genes in our species. A semi-extensive PCR approach involving *A. minutum *DNA and primers designed for the cyanobacterial genes also failed to detect congruent sequences related to PSP toxin biosynthesis. Although EST libraries such as the one presented here represent only a subsample of the transcriptome, the lack of evidence for all of these genes in our library, as well as in other published EST libraries of PSP toxin-producing *Alexandrium *species, suggests that the PSP toxin genes in dinoflagellates are more different from their cyanobacterial counterparts than would be expected in the case of a recent gene transfer. This does not rule out a cyanobacterial ancestry for the dinoflagellate PSP toxin genes, because the possibility of rapid change of newly obtained genes in the dinoflagellate nucleus cannot be excluded.

Microarray-based comparisons of toxic and non-toxic strains of *A. minutum *indicated that many genes were higher expressed in the toxic than in the non-toxic strain. Among contigs candidates for PSP toxin-related genes are the several genes that could not be annotated due to a lack of known similar sequences but were expressed only in the toxic strains. Further testing of these candidates, by expression analysis with different toxic and non-toxic strains and by physiological manipulations that affect the biosynthetic rate and cell content of particular toxin analogues is clearly warranted.

While transformation of dinoflagellates has rarely been achieved [84], several studies have demonstrated successful expression of dinoflagellate genes in *E. coli *[85,86] or yeast [87,88]. To obtain conclusive evidence for the function of PSP toxin genes it might be necessary to use heterologous expression of the identified candidates in a suitable expression system, followed by purification of the enzymes and assessment of their activity on known PSP toxin precursors.

## Authors' contributions

UJ and AC conceived of the study, participated in its design and helped to draft the manuscript; UJ also participated in the day-to-day management of this study and in the design of the microarray. GG sequenced the cDNA library, constructed the EST contigs and provided part of the bioinformatic analysis. BK conceived of the LC-MS/MS measurement protocol, carried out these measurements and provided the LC-FD protocols. AG provided the SAMS part of the automated annotation. IY and SB determined the growth conditions, grew and harvested the input cultures for the EST library. SB conceived of the RNA extraction protocol. IY carried out the remainder of the molecular genetics studies, participated in the bioinformatics analysis, carried out the manual annotation and drafted the manuscript. All authors read and approved the final paper.

## Supplementary Material

Additional file 1 **Primer tables and respective PCR conditions**. All primers used and the respective PCR protocols of the study are listed in this file. Click here for file

Additional file 2 **Phyml-based likelihood trees with bootstrap support values**. 14 phylogenies of *A. minutum *EST contigs that produced significant (e < 10^-4^) BLAST hits with cyanobacterial sxt-related genes. Phylogenies were calculated including the *A. minutum *contig sequence, the corresponding cyanobacterial gene, and their closest SwissProt matches as identified by PhylogGena (Top10Select-mode). Phylogenies M.2 and N.2 additionally include the best 20 hits produced by blasting the *Alexandrium *sequences against the NCBI non-redundant protein sequence database (nr). Click here for file

Additional file 3 **List of differentially expressed genes**. Table of contigs identified as differentially expressed between both toxic strains and the non-toxic strain at two time-points during light phase. Included are contig IDs, microarray-based gene expression data, characterisation of the contigs, manual annotation and results of various database searches. Click here for file

Additional file 4 **Rarefaction curve**. Generated ESTs and assembled cluster as contigs were analysed using http://www.biology.ualberta.ca/jbrzusto/rarefact.php#Calculator. Click here for file
