# Supplementary material for: Comparative gene expression in toxic versus non-toxic strains of the marine dinoflagellate Alexandrium minutum
Source: BMC Genomics. 2010 Apr 19;11:248. doi: 10.1186/1471-2164-11-248 (PMC2874808; doi:10.1186/1471-2164-11-248)
Supplement: Additional file 1 — Primer tables and respective PCR conditions. All primers used and the respective PCR protocols of the study are listed in this file. [file 1471-2164-11-248-S1.PDF]

Primers for cyanobacterial sxt gene cluster

gene	Amplicon size (nt)	primer sequence 5'-3'	tm
sxtG	675	sxtGF GATTTACCAATCGCCAAGCA	53.6
		sxtGR GTAACCATTCTATGCCCAAACC	53.9
sxtU	690	sxtUF ATTGGATGGAAAAGTGGCGATTA	54.9
		sxtUR TGATCGGGTTGTGTCACTGCATA	58.5
sxtF	762	norM8F ATCTGATATTAGAAGCACGAGCA	53.7
		norM8R CGTTAAATAGTGCCGTTTCCG	54.3
sxt15	601	sxt15F GGTACTATGGCGCAGTTACGA	56.7
		sxt15R TAGCAGTTTCGGAGGATTTC	52.2
sxt14	481	sxt14F TTTTGACTCAGCAGGTAATG	50.3
		sxt14R GAATTGGTTTGGAAGGATTT	48.7
sxtX	645	sxtXF GAGAAGATAAATGTGCTATT	44.8
		sxtXR GAGCCGTTGTAACCGTA	51.7
sxtA	680	sxtA1F TAATCGTTATACTCACGGCTTTG	52.7
(ACP domain)		sxtA1R AAGCATCTCTTTGGAATACGG	52.7
sxtA	740	sxtA3F CAAGGTCGCAAGGTGCTAA	55.4
(Aminotransferase domain)		sxtA3R CCTCCTGCTTACAAGGCAAA	55.3
sxtH	660	sxtHF AAACCAGGAAGTATCACCACGGC	59.4
		sxtHR GCATCAAATCCGCAGTCCG	56.9
sxtI	714	sxtIaF CTACCACGATAGTGCTGCCG	57.8
		sxtIaR CCCCACCGTGTAGTTGAAGT	57
sxtT	662	sxtTF GCACGTAGTCGCCAATGTAGAAG	57.8
		sxtTR GGGGATTCTGAAACGCAGTATTG	56.1
sxtM	770	norM16F ATGATGGGCTTACTTGGTACTCA	55.7
		norM16R CCCGATTCCAAGAGGTATCACT	56.1
sxtN	347	sxtNF TACCCGTGCTTTTGAGAACTTAG	54.8
		sxtNR CCTGTCTTTCTCTCCGCTATA	53.3
sxtO	446	sxtOF AATTAGAGCAGACTTTGTTC	47.8
		sxtOR TGTTTTCCACGTATTTG	44.4

Gradient a: 6 temperature steps, approximately 48 – 58 °C;
Gradient b: 6 temperature steps, approximately 48.2 – 58.5 °C

30 µl reactions:

- 20 ng genomic DNA
- 0.2 µmol l^{-1} forward and reverse primers
- 0.2 mmol l^{-1} dNTPs

- HotMasterTaq buffer 1X (Eppendorf)
- 1.5 units of HotMaster *Taq* DNA polymerase (Eppendorf)

sxtO: 5 temperature steps, approximately 46 - 53.5 °C; 2 DNA concentrations at 46°C: 33 ng DNA, 100 ng DNA

Cycling conditions were based on those optimal for these primers (K.Stucken, pers. comm.).: 94°C for 5 min, 20 cycles with denaturation 94°C (20 s), annealing for 45 s, extension at 70°C (30 s), and a final extension step of 10 min at 70°C. PCR products were visualized using agarose gel electrophoresis.

M13-primer PCR for amplification of cDNA plasmid inserts to use in calibration curves:

50 µl reactions:
- 0.5 µl plasmid stock
- 0.2 µmole l^{-1} forward and reverse primers
- 0.2 mmole l^{-1} dNTPs
- HotMasterTaq buffer 1X (Eppendorf)
- 2.5 units of HotMaster *Taq* DNA polymerase (Eppendorf)

Cycling conditions:

Cycling conditions: 94°C for 2 min, 24 cycles with denaturation 94°C (1 min), annealing for 1 min, extension at 72°C (2 min), and a final extension step of 10 min at 72°C. PCR products were visualized using agarose gel electrophoresis.

qPCR

primers: 5‘-3‘

```
Amin_44h03_406F   ACAAGGTCAGAATGCGGAAGA

Amin_44h03_505r   GCAGCAACAGAGCCCATGT

Amin_52d01_503F   GAGGAGAGCTGCGACCACAT

Amin_52d01_602r   CAAGTTGGGAGTCACATTTCCA

Amin_56a03_417F   CAGCACGAACGCACCTTGTA

Amin_56a03_516r   TTGACGTGGATTGCCTGAGA

Amin_81i24_193F   ATGCGGAGCCTGTTGAGATC
```

Amin_81i24_292r	GTAGCTCCAGTGCGCAAGTG
Amin_88h09_564F	ACAGTGAGCCAGCGAGTGAA
Amin_88h09_663r	TCCATGCTCTGCCAATCTTG
Amin_89d06_358F	CGAACCCGAACTGGAAGGT
Amin_89d06_457r	TCATCGCCTACCCGGTACAC

cDNA synthesis

cDNA was synthesised using SuperScript III reverse transcriptase (Invitrogen, Karlsruhe, Germany) according to the manufacturer's instruction. Each reaction contained 500 ng total RNA supplemented with the artificial control RNAs (1 ng $reaction^{-1}$) and NSP (1 pg $reaction^{-1}$) and 50 pmol oligo dT primer.
