# Supplementary figures and images for: Comparative gene expression in toxic versus non-toxic strains of the marine dinoflagellate Alexandrium minutum
Source: BMC Genomics. 2010 Apr 19;11:248. doi: 10.1186/1471-2164-11-248 (PMC2874808; doi:10.1186/1471-2164-11-248)

A

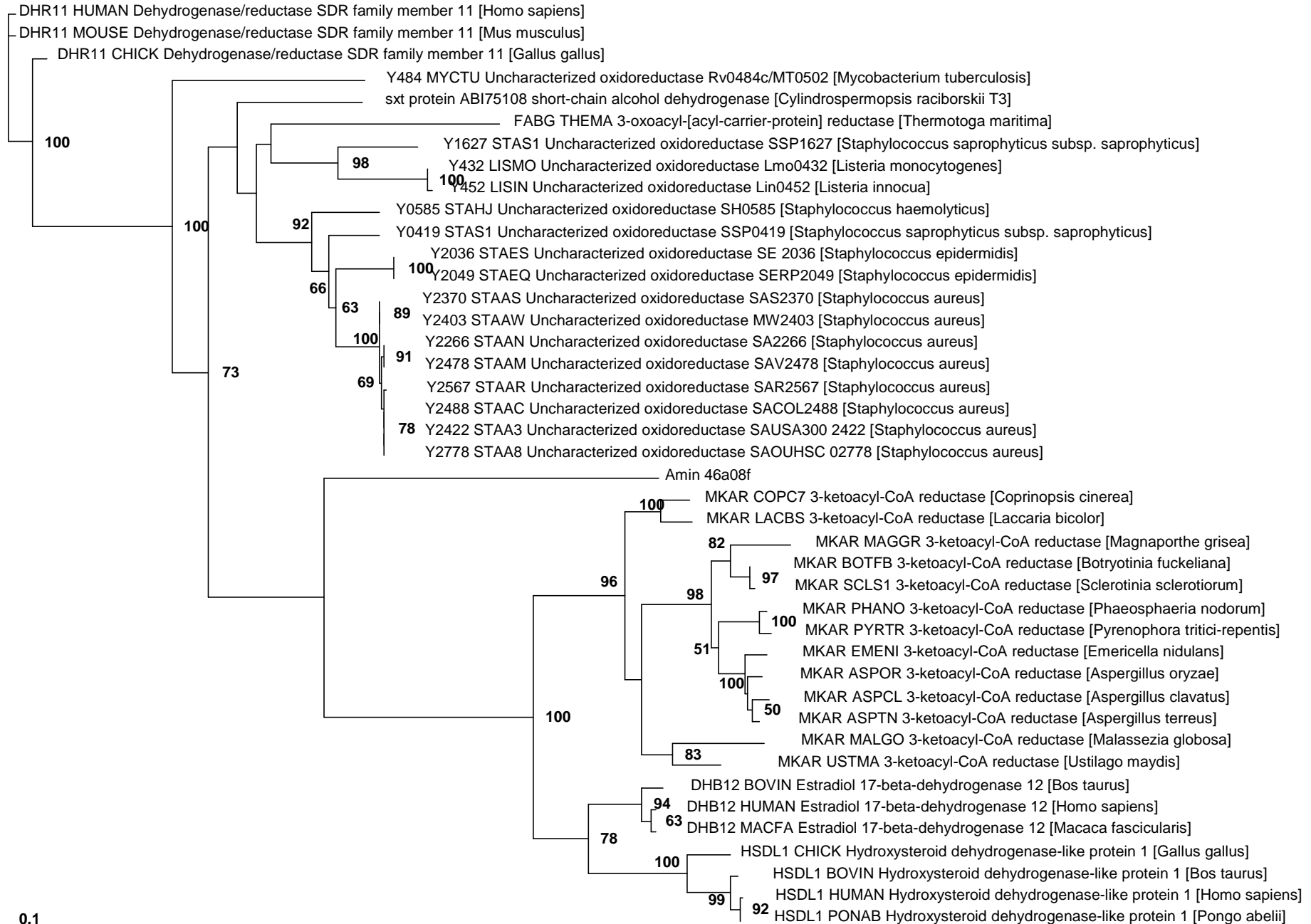

**B**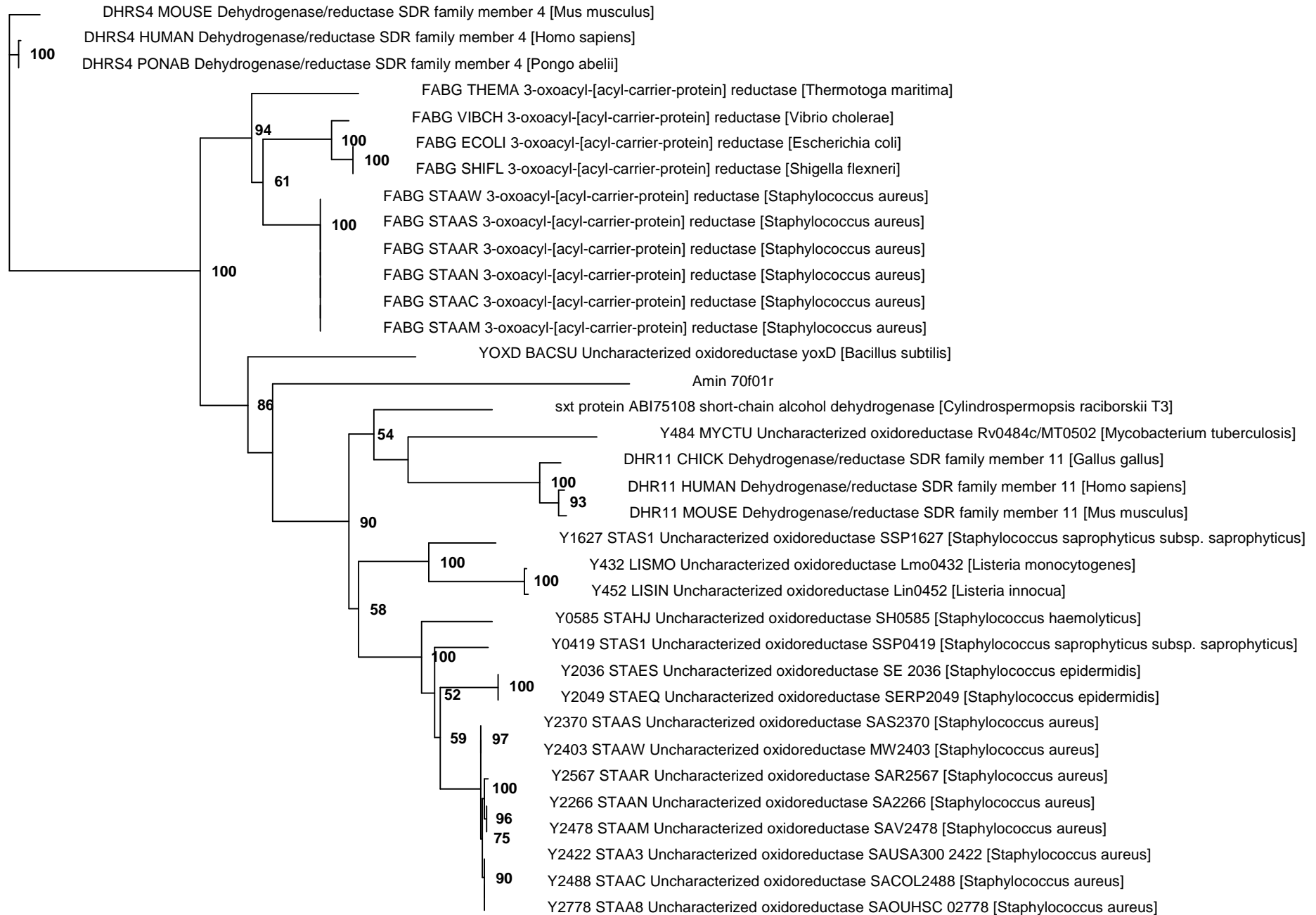

C

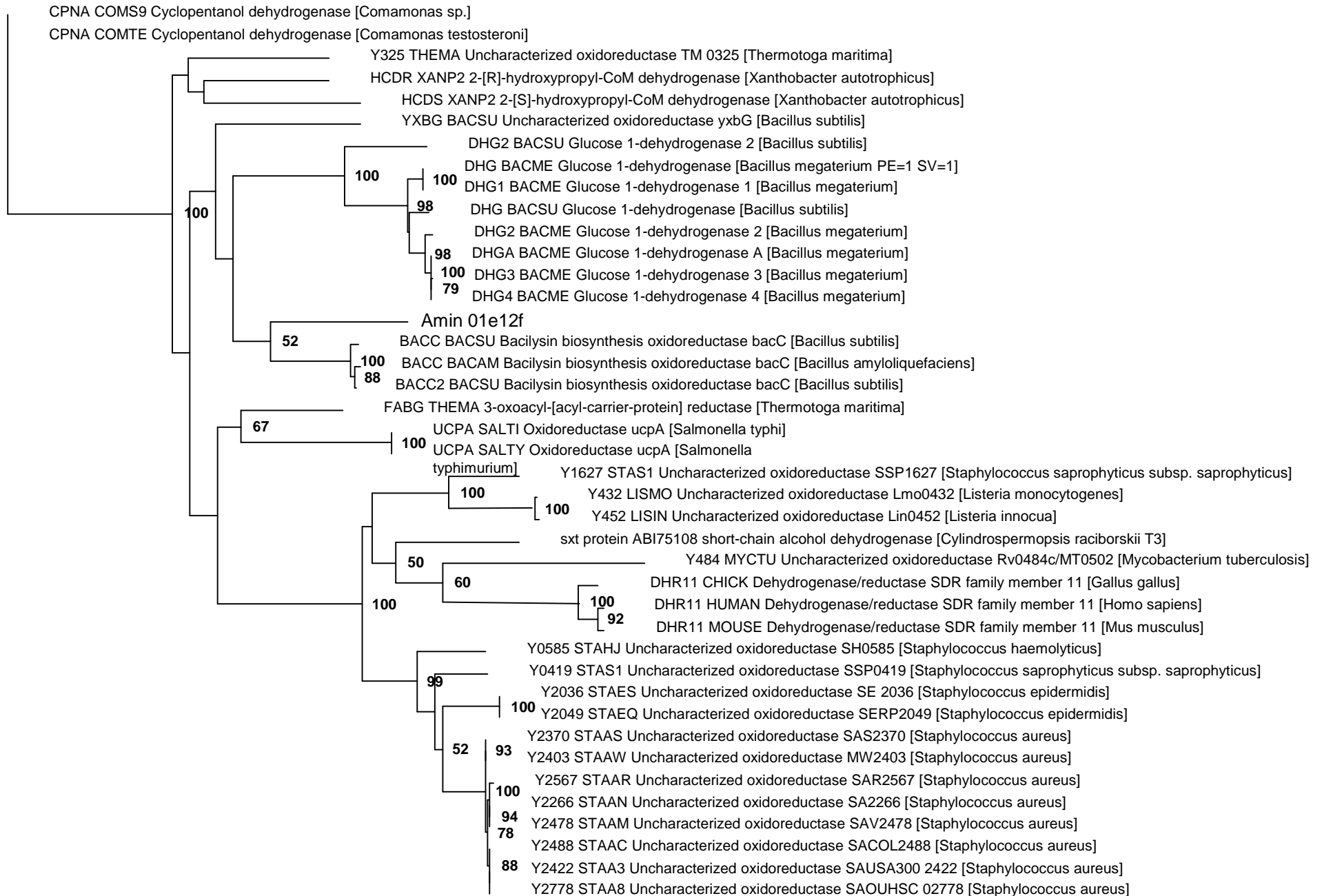

D

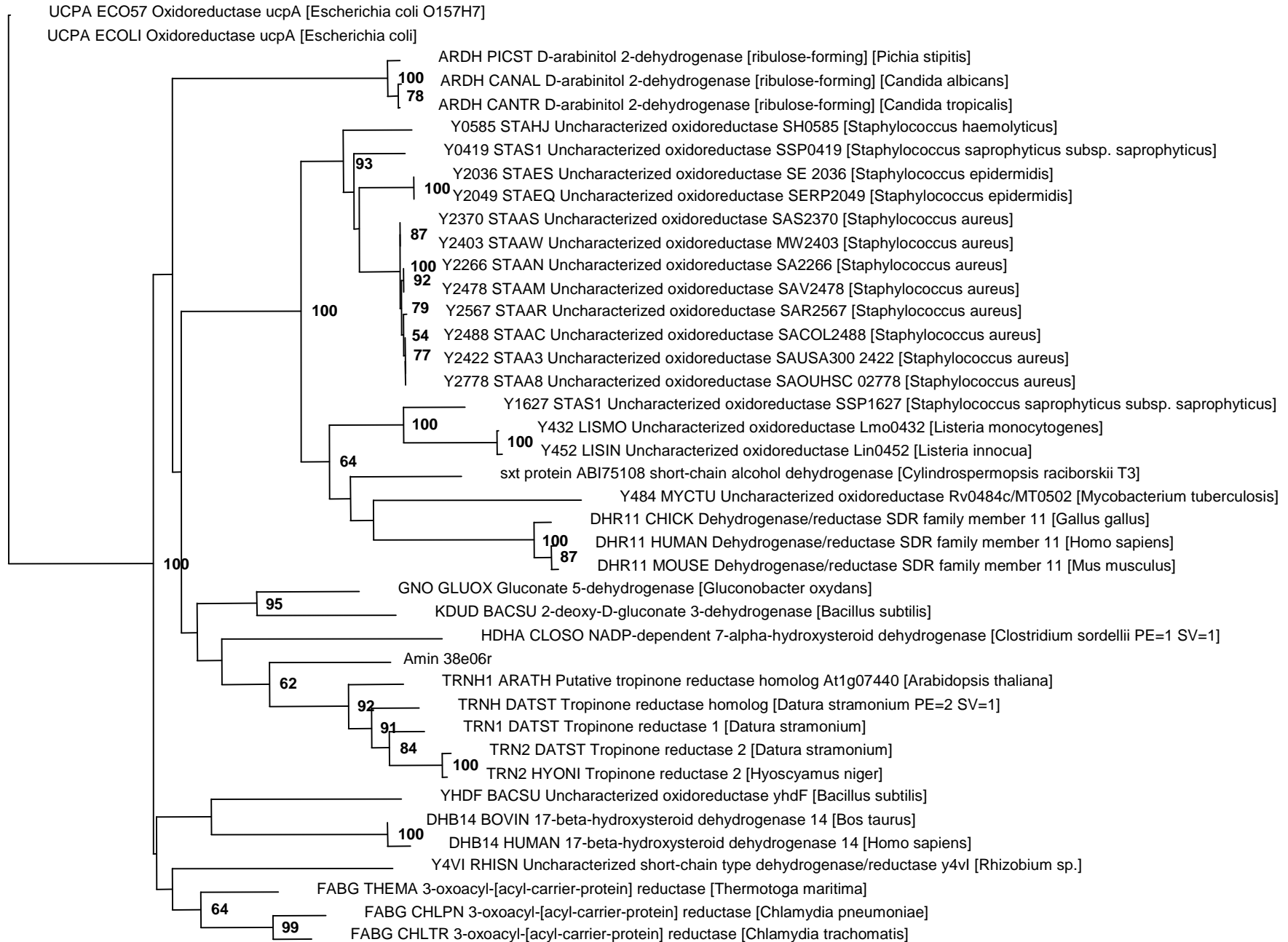

E

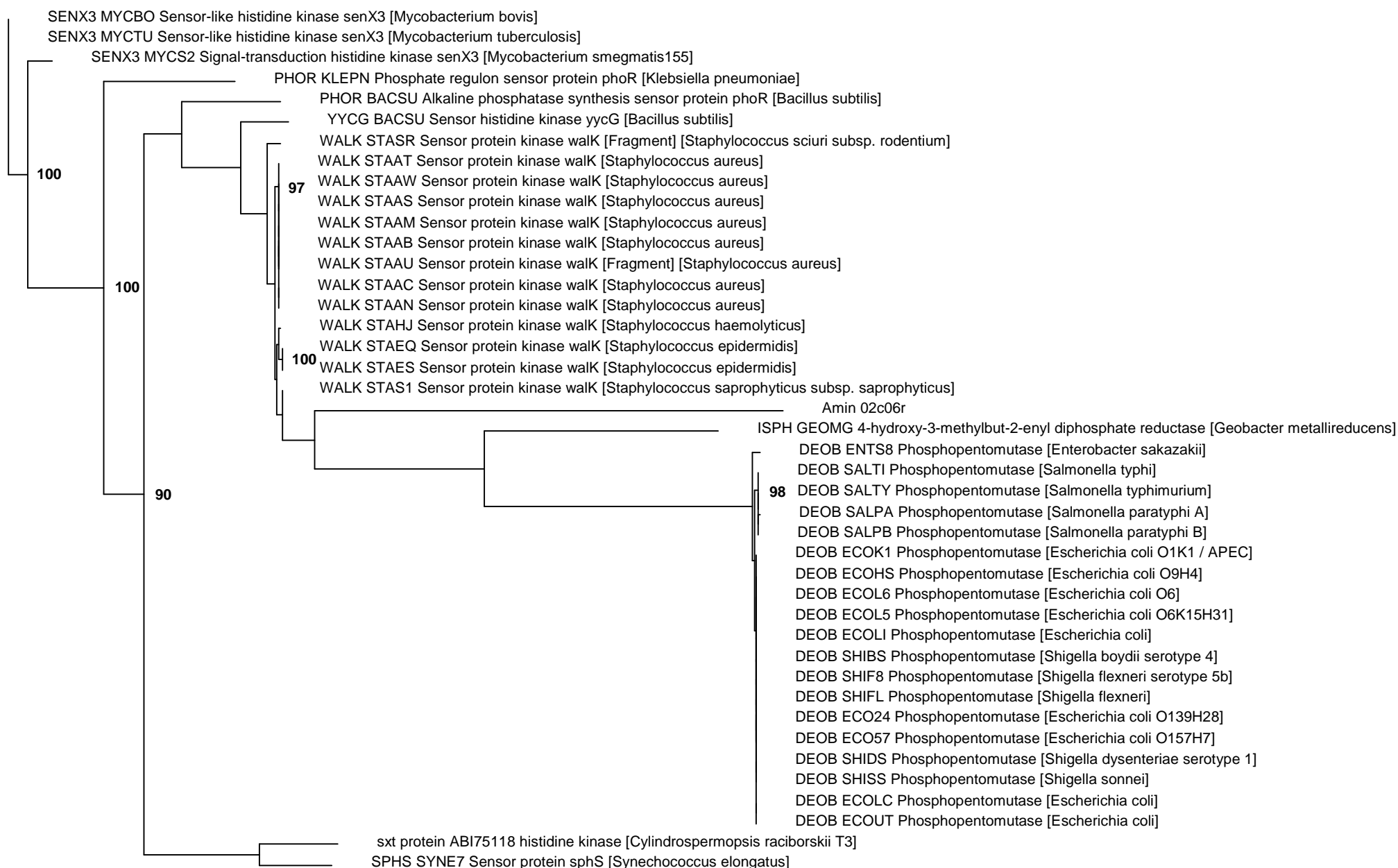

F

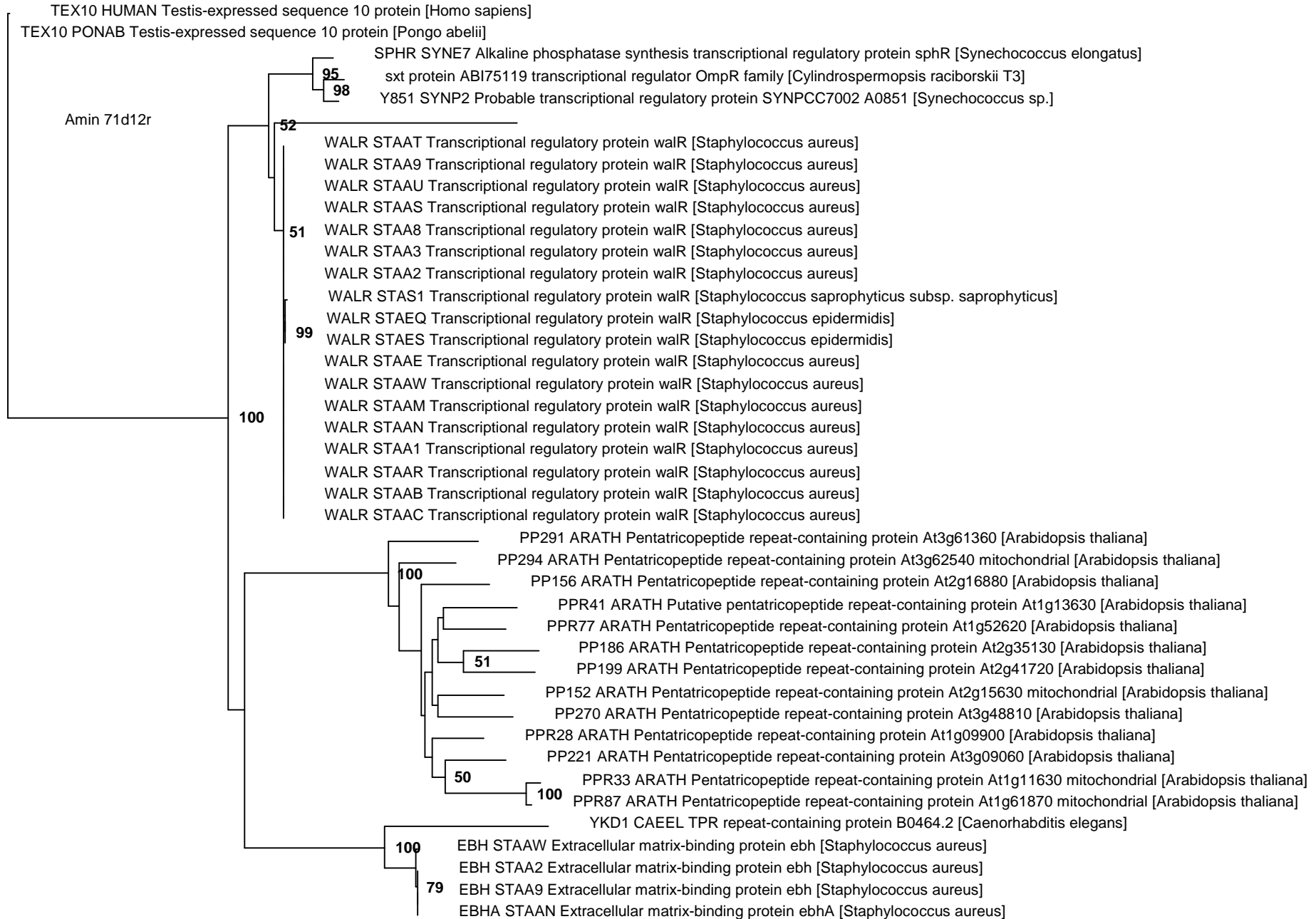

G

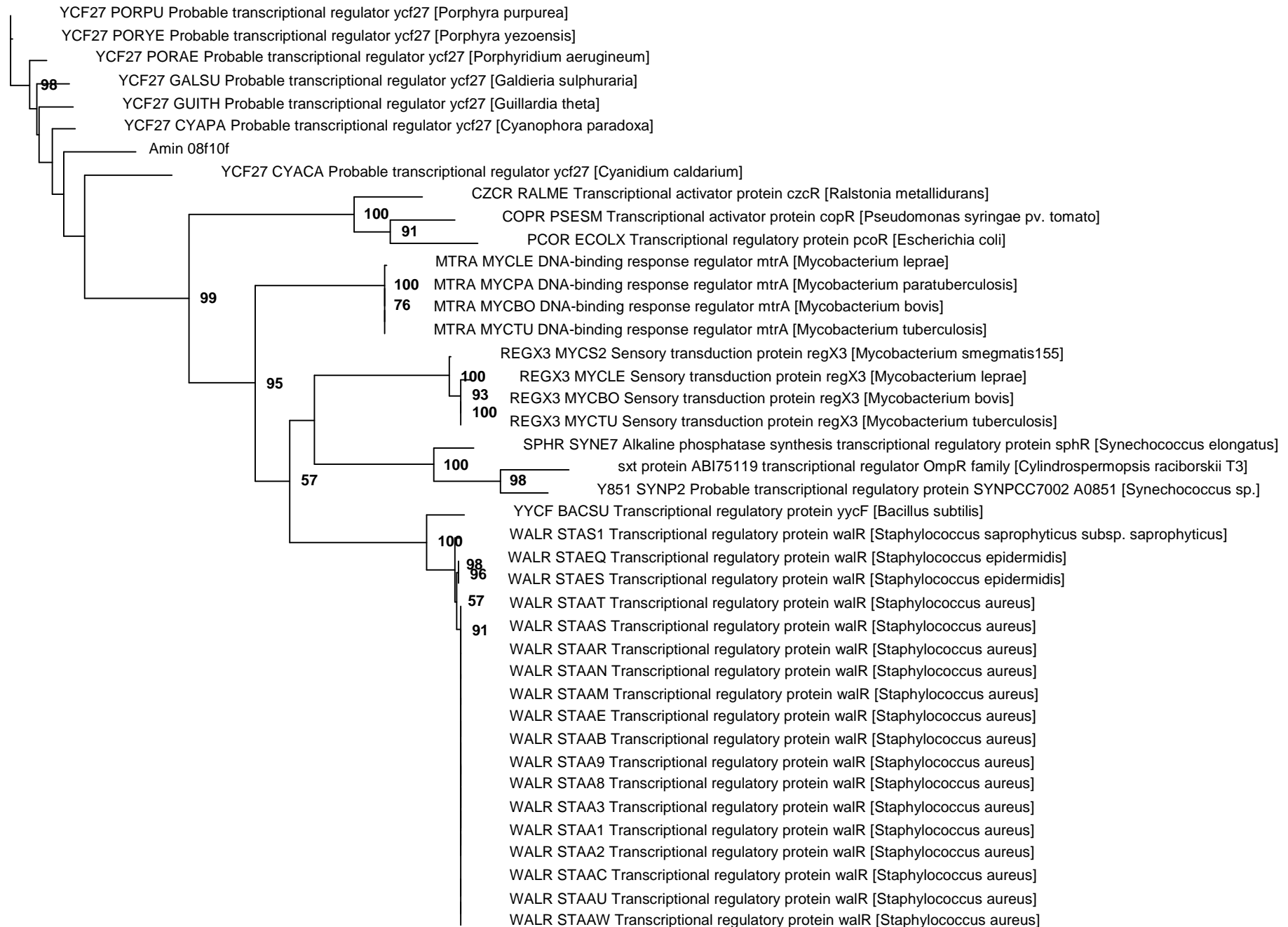

H

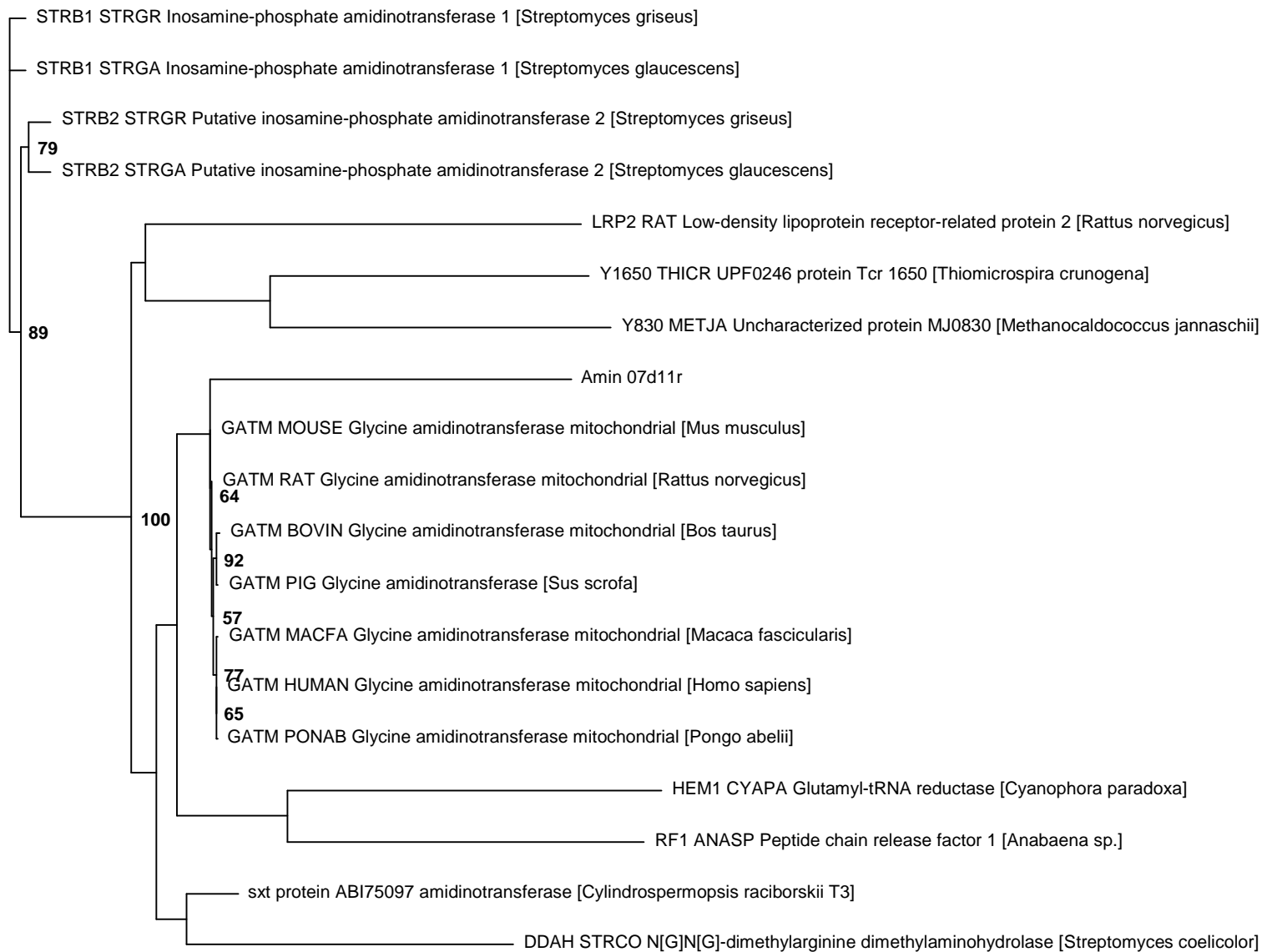

\_0.1

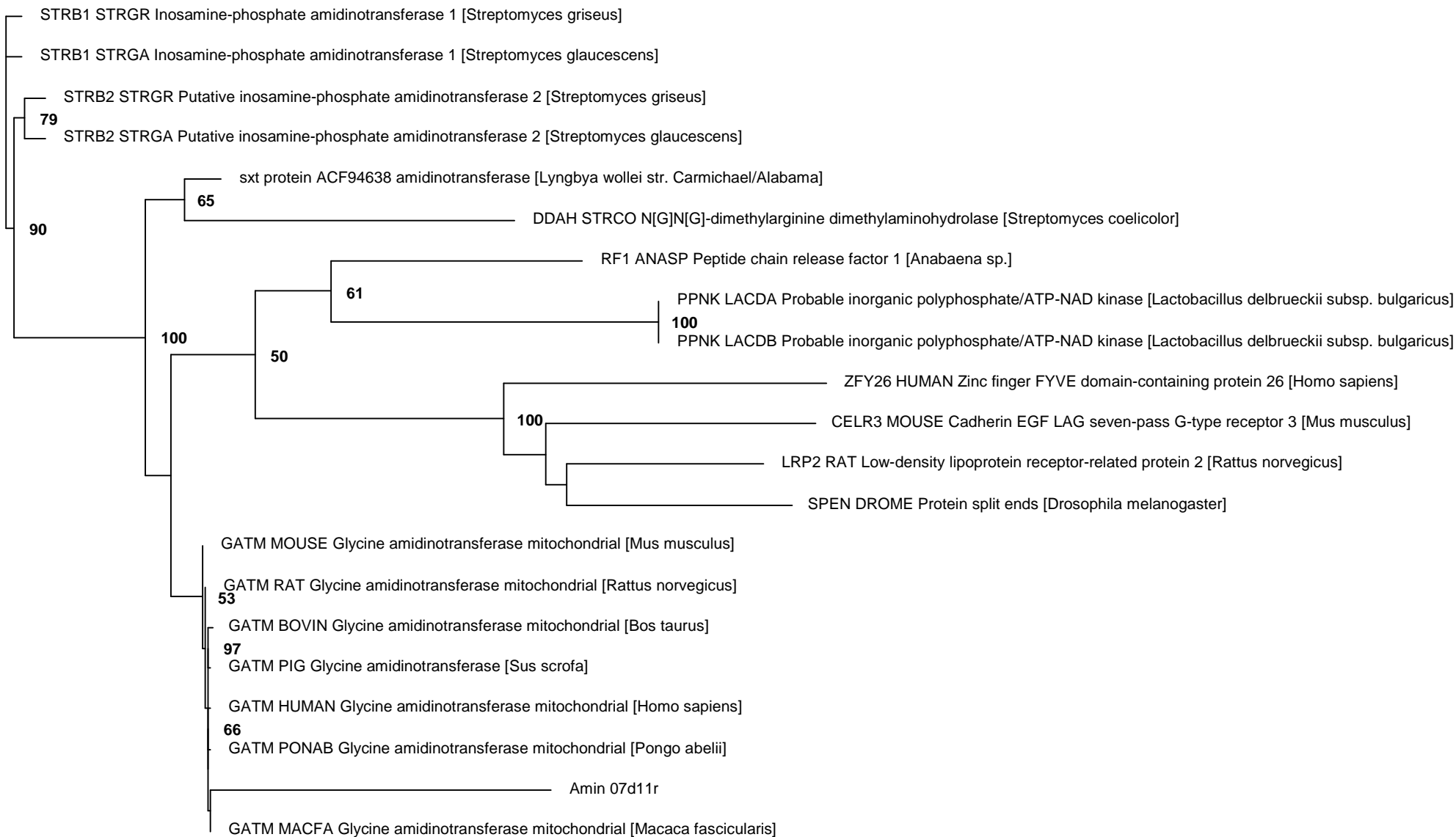

J

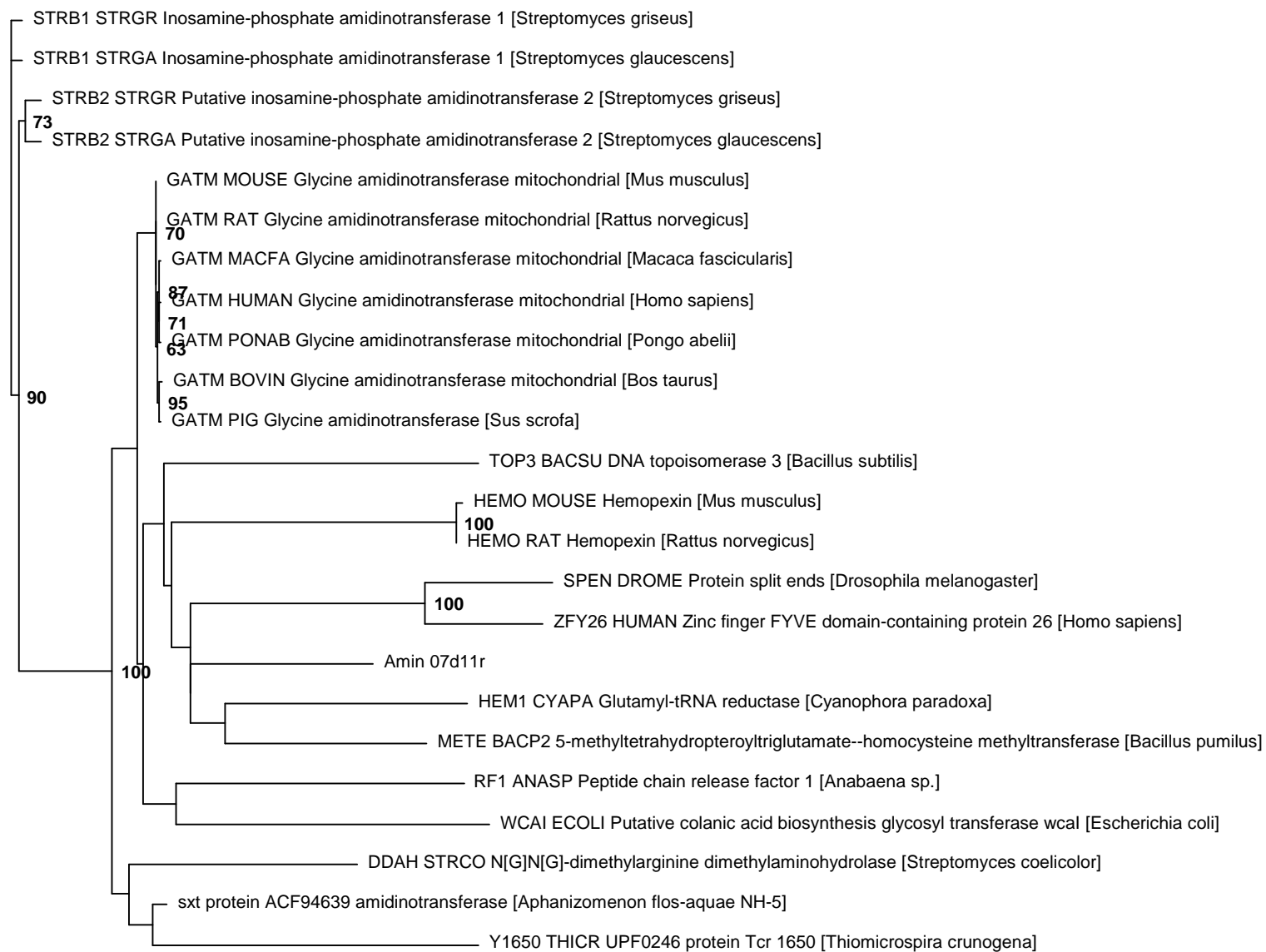

\_0.1

K

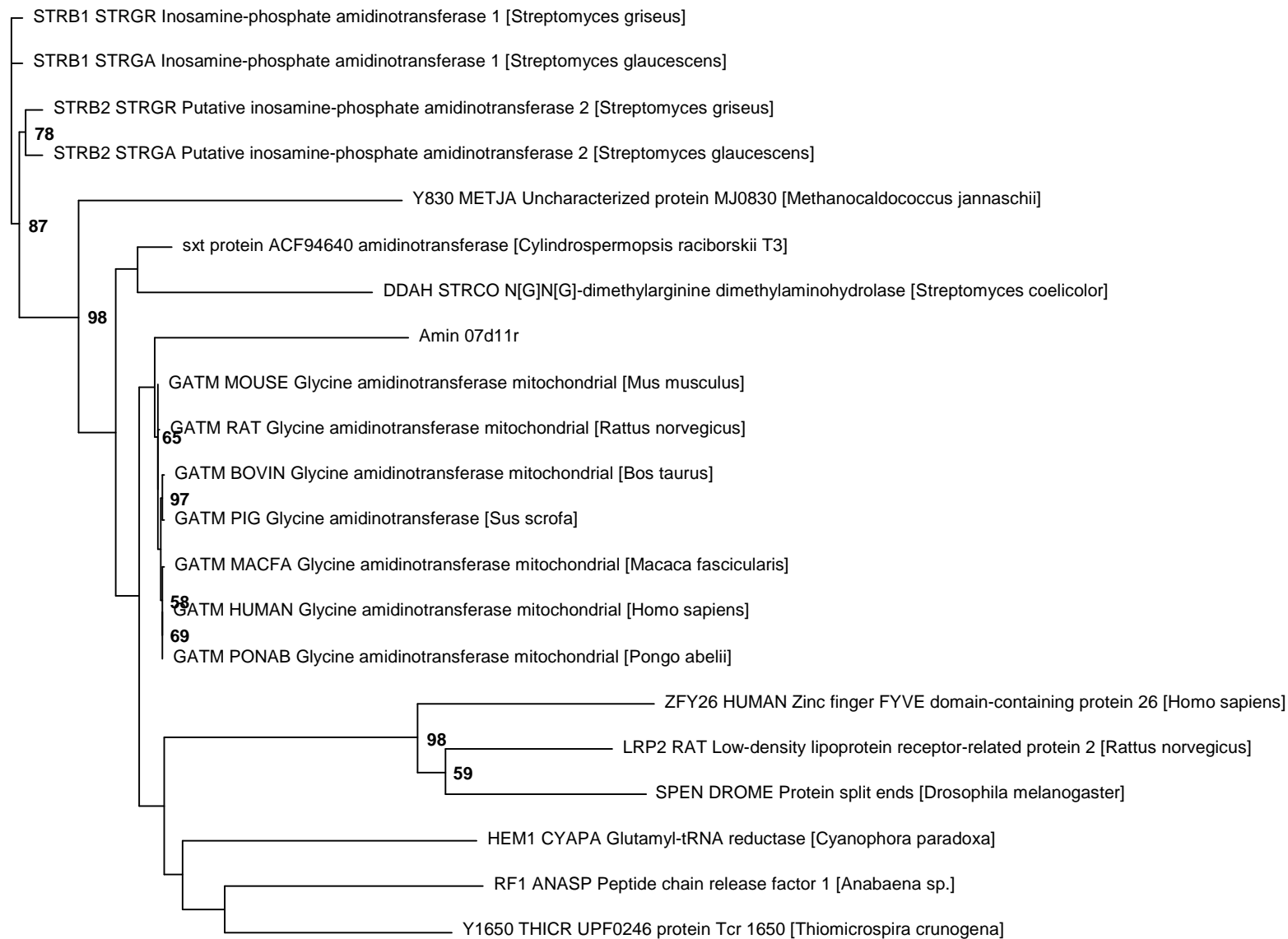

\_0.1

L

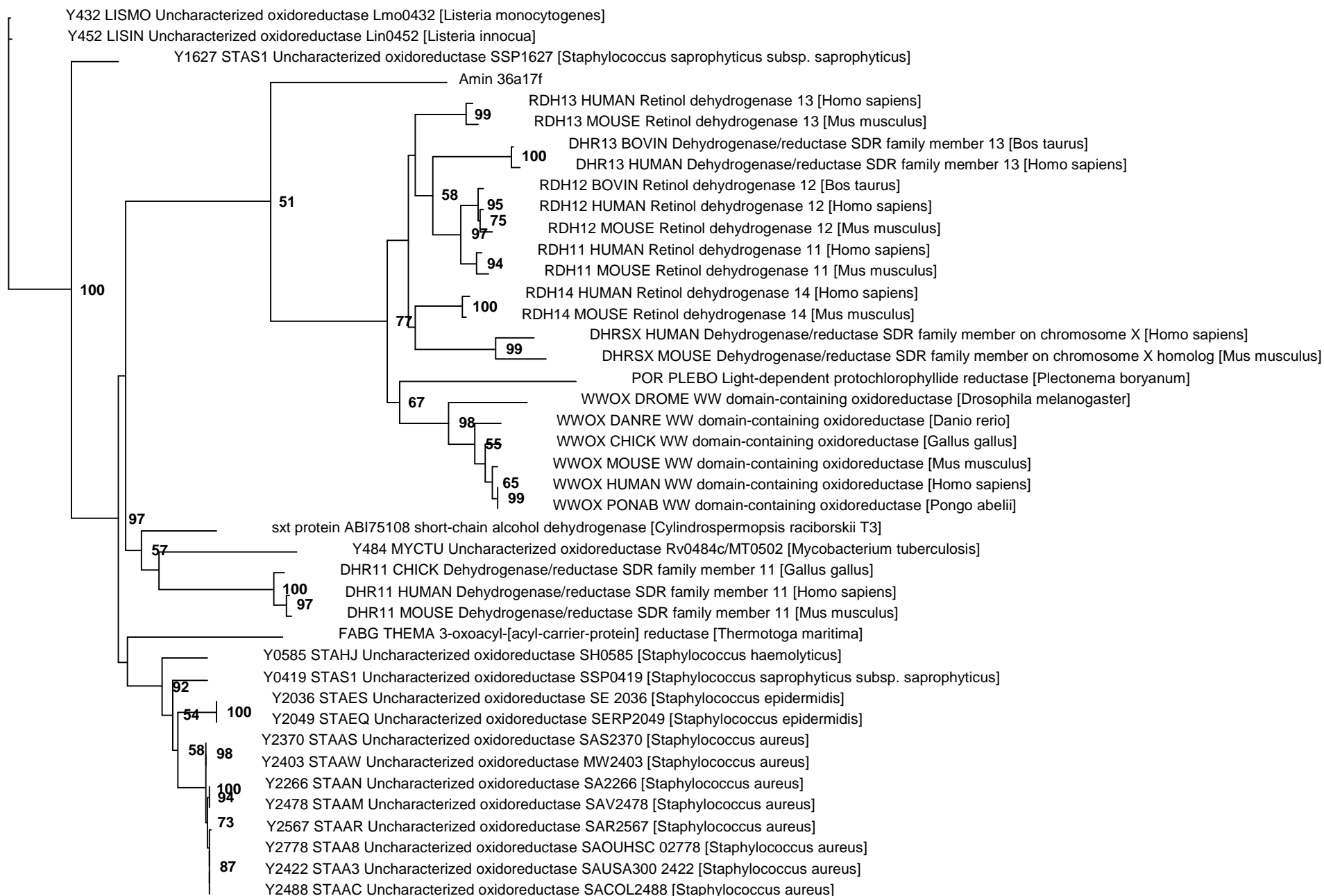

M.1

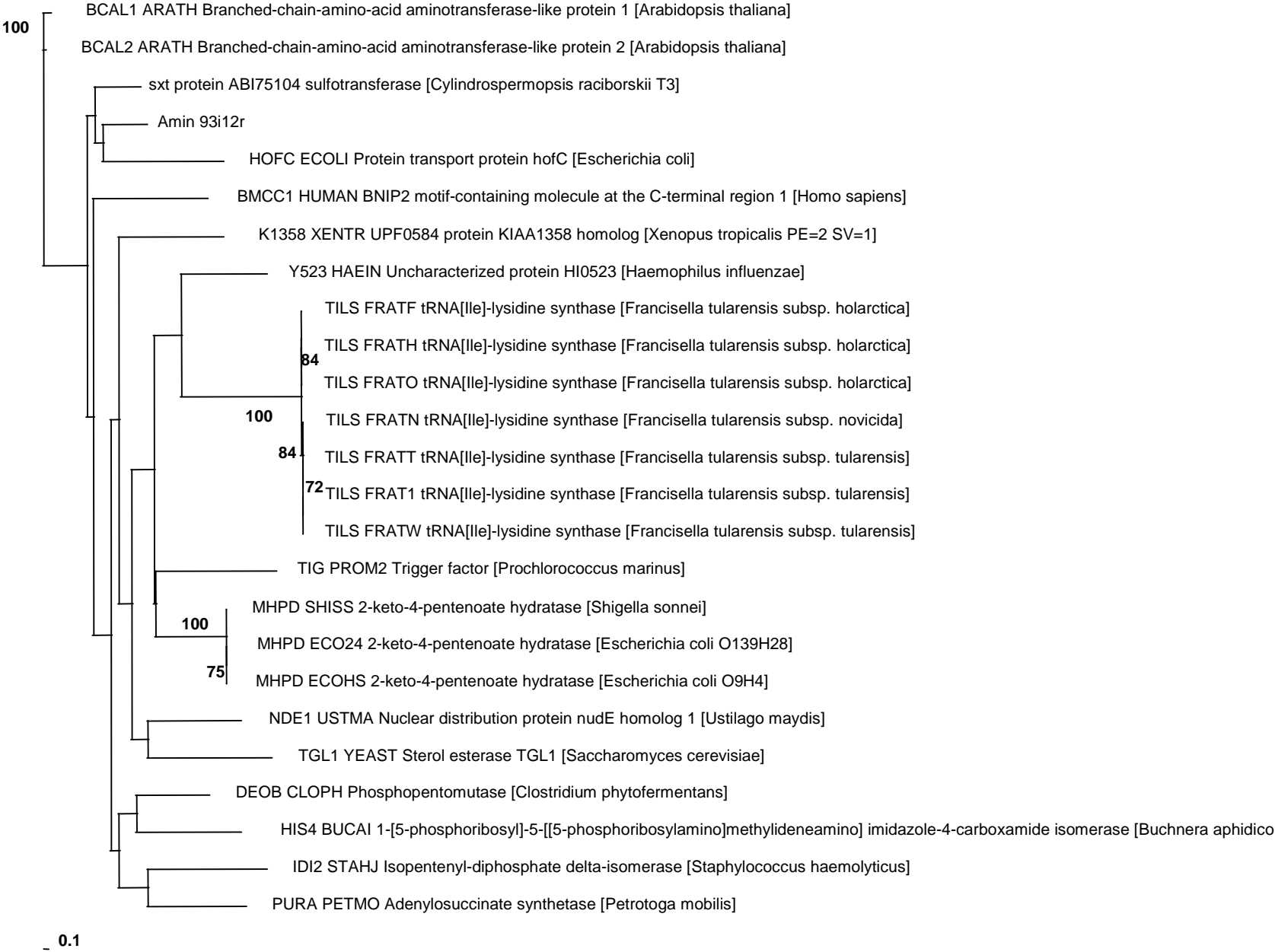

# M.2

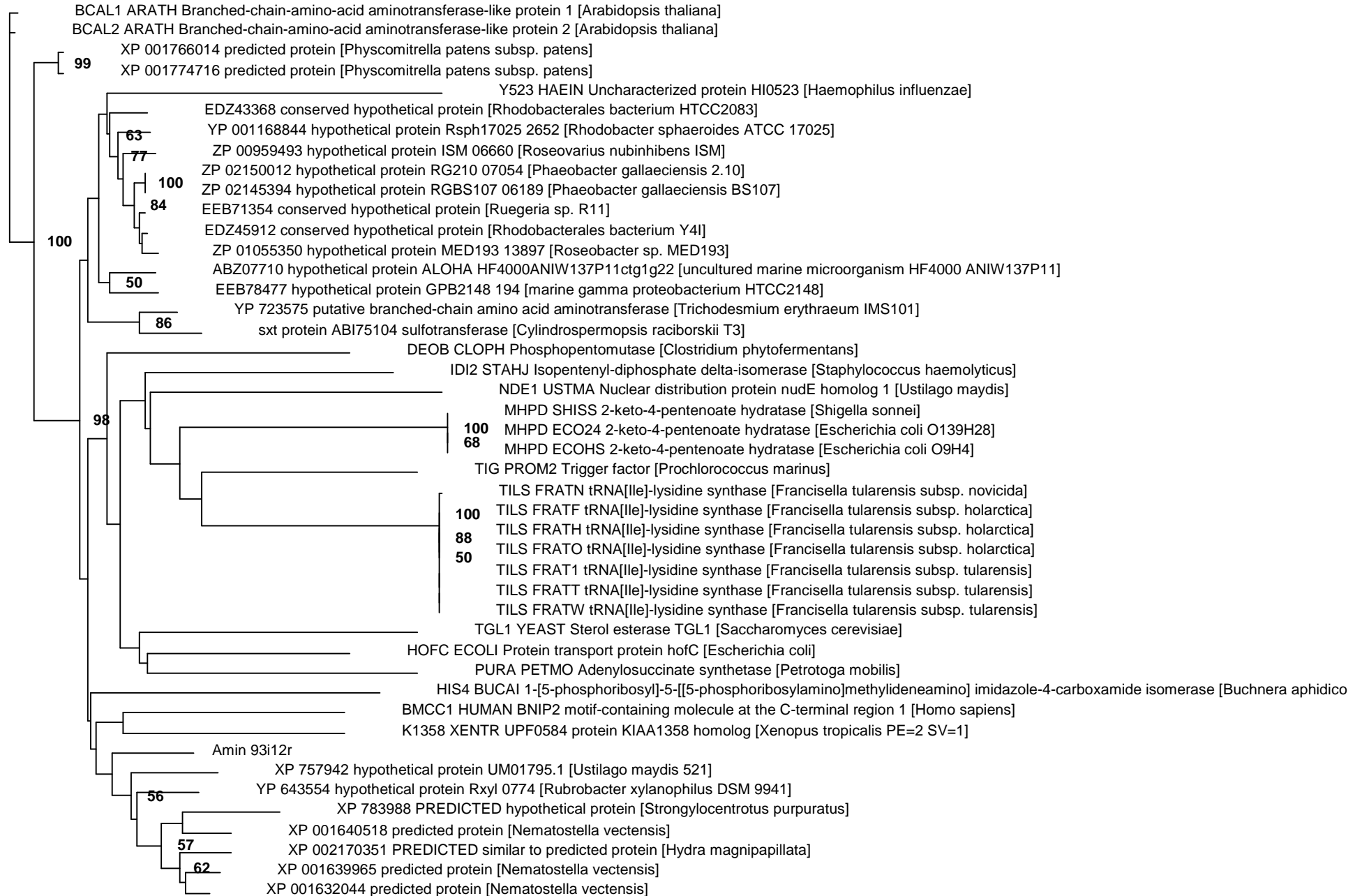

# N.1

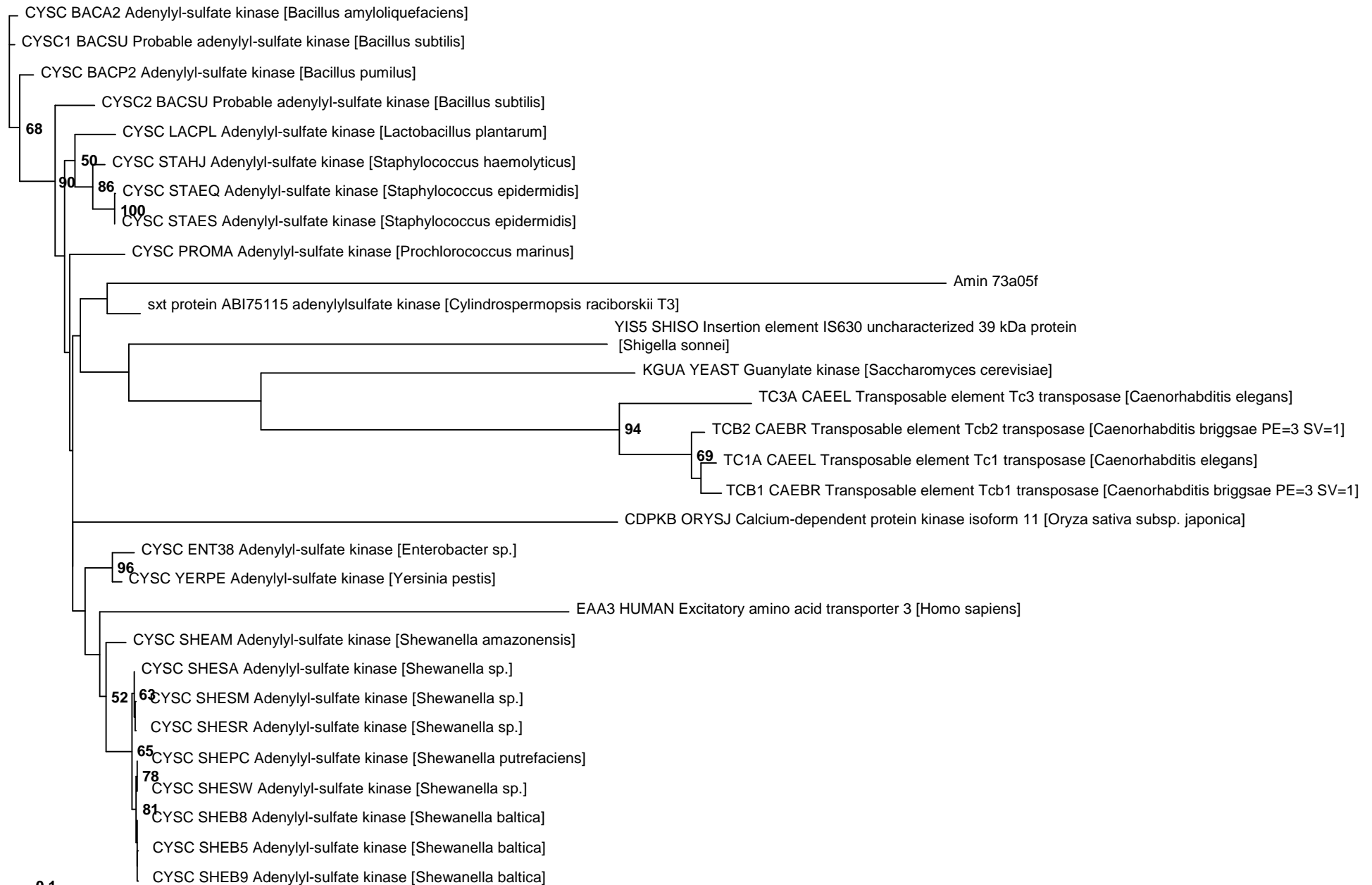

# N.2

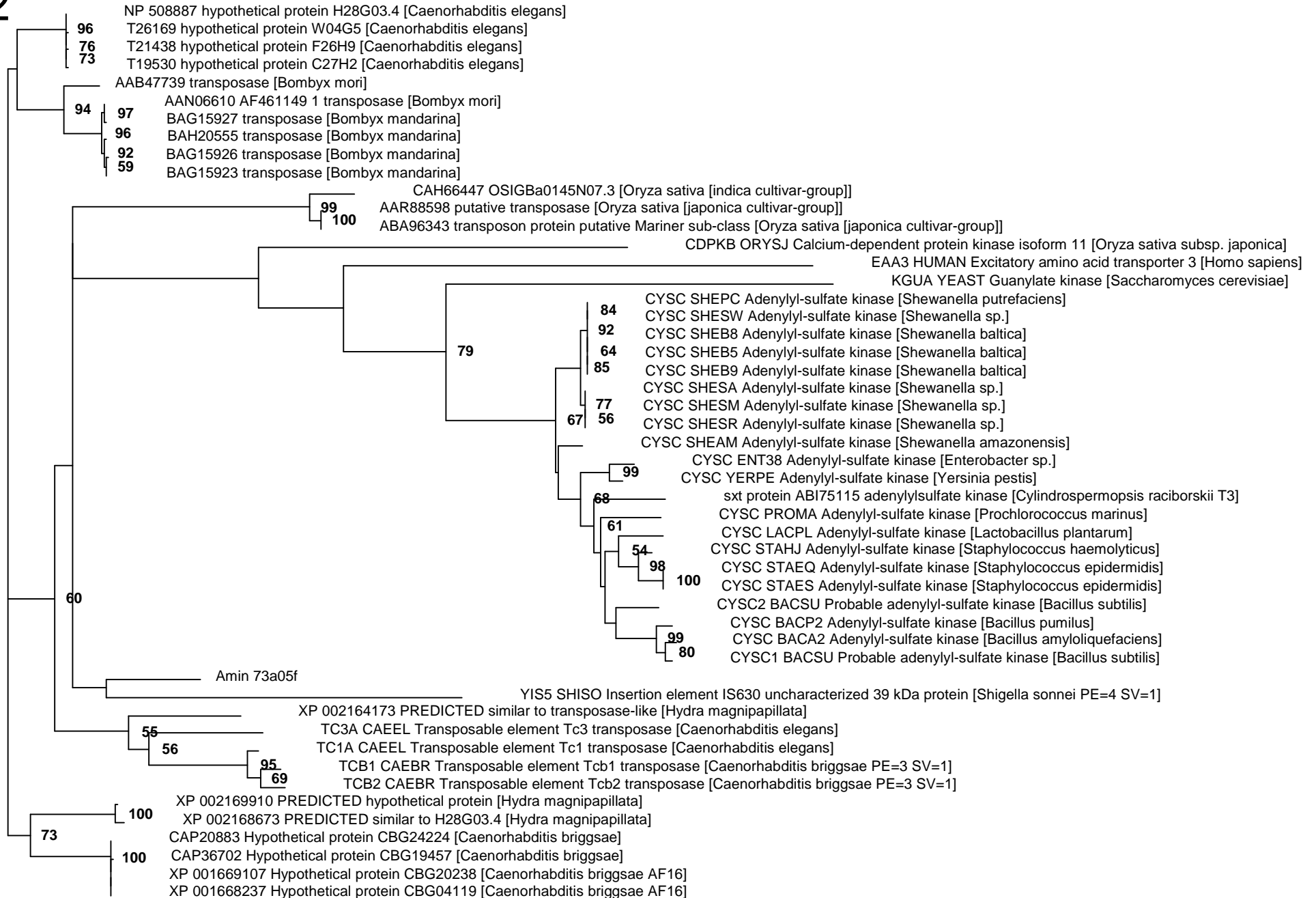

0.1

Supplement: Additional file 2 — Phyml-based likelihood trees with bootstrap support values. 14 phylogenies of A. minutum EST contigs that produced significant (e < 10-4) BLAST hits with cyanobacterial sxt-related genes. Phylogenies were calculated including the A. minutum contig sequence, the corresponding cyanobacterial gene, and their closest SwissProt matches as identified by PhylogGena (Top10Select-mode). Phylogenies M.2 and N.2 additionally include the best 20 hits produced by blasting the Alexandrium sequences against the NCBI non-redundant protein sequence database (nr). [file 1471-2164-11-248-S2.PDF]

## rarefaction curve for Alexandrium

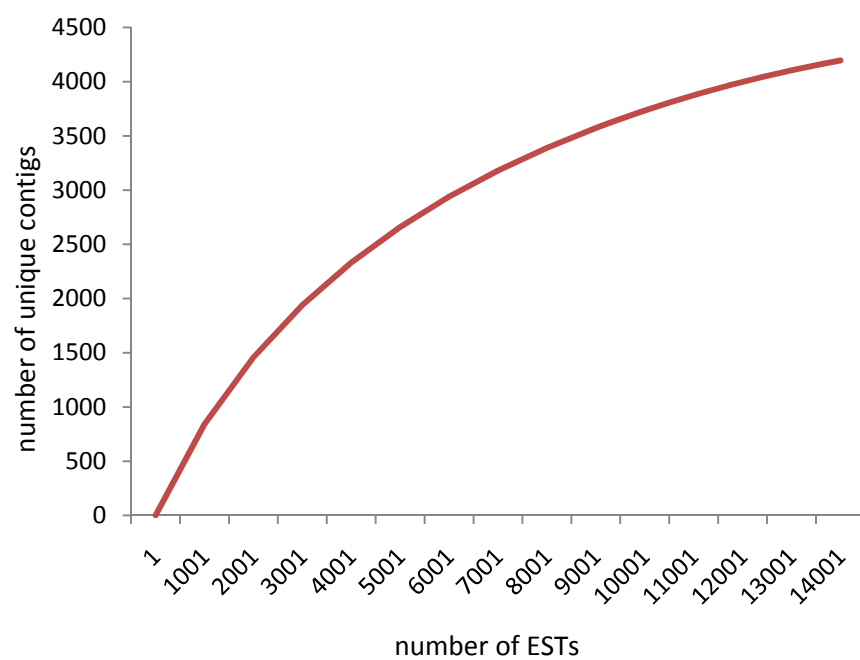

Supplement: Additional file 4 — Rarefaction curve. Generated ESTs and assembled cluster as contigs were analysed using http://www.biology.ualberta.ca/jbrzusto/rarefact.php#Calculator. [file 1471-2164-11-248-S4.PDF]
